# Integrating Microbial Community Data Into an Ecosystem‐Scale Model to Predict Litter Decomposition in the Face of Climate Change

**DOI:** 10.1111/gcb.70352

**Published:** 2025-07-17

**Authors:** Katherine S. Rocci, Derek Pierson, Fiona V. Jevon, Alexander Polussa, Angela M. Oliverio, Mark A. Bradford, Peter B. Reich, William R. Wieder

**Affiliations:** ^1^ Institute of Arctic and Alpine Research University of Colorado Boulder Colorado USA; ^2^ Institute for Global Change Biology University of Michigan Ann Arbor Michigan USA; ^3^ Rocky Mountain Research Station United States Forest Service Boise Idaho USA; ^4^ The Forest School Yale School of the Environment, Yale University New Haven Connecticut USA; ^5^ Biology Department Syracuse University Syracuse New York USA; ^6^ Department of Forest Resources University of Minnesota St. Paul Minnesota USA; ^7^ Climate and Global Dynamics Laboratory National Center for Atmospheric Research Boulder Colorado USA

**Keywords:** climate change impacts, data‐model integration, ecosystem‐scale model, litter decomposition, litterbags, microbial traits, model calibration, soil microbial communities

## Abstract

Litter decomposition is an important ecosystem process and global carbon flux that has been shown to be controlled by climate, litter quality, and microbial communities. Process‐based ecosystem models are used to predict responses of litter decomposition to climate change. While these models represent climate and litter quality effects on litter decomposition, they have yet to integrate empirical microbial community data into their parameterizations for predicting litter decomposition. To fill this gap, our research used a comprehensive leaf litterbag decomposition experiment at 10 temperate forest U.S. National Ecological Observatory Network (NEON) sites to calibrate (7 sites) and validate (3 sites) the MIcrobial‐MIneral Carbon Stabilization (MIMICS) model. MIMICS was calibrated to empirical decomposition rates and to their empirical drivers, including the microbial community (represented as the copiotroph‐to‐oligotroph ratio). We calibrate to empirical drivers, rather than solely rates or pool sizes, to improve the underlying drivers of modeled leaf litter decomposition. We then validated the calibrated model and evaluated the effects of calibration under climate change using the SSP 3–7.0 climate change scenario. We find that incorporating empirical drivers of litter decomposition provides similar, and sometimes better (in terms of goodness‐of‐fit metrics), predictions of leaf litter decomposition but with different underlying ecological dynamics. For some sites, calibration also increased climate change‐induced leaf litter mass loss by up to 5%, with implications for carbon cycle‐climate feedbacks. Our work also provides an example for integrating data on the relative abundance of bacterial functional groups into an ecosystem model using a novel calibration method to bridge empiricism and process‐based modeling, answering a call for the use of empirical microbial community data in process‐based ecosystem models. We highlight that incorporating mechanistic information into models, as done in this study, is important for improving confidence in model projections of ecological processes like litter decomposition under climate change.

## Graphical abstract

Representing observational drivers of ecological processes like litter decomposition in computer models is important for improving confidence in climate change predictions. We calibrate an ecosystem‐scale model to observed soil moisture, litter quality, and microbial community (the ratio of fast to slow‐growing bacteria) data as drivers of temperate forest leaf litter decomposition. The calibrated model provided better alignment with observational drivers without reducing traditional model performance metrics and also predicted faster litter decomposition under climate change at 3 of 7 sites, providing improved confidence in model output with implications for carbon‐climate feedbacks.

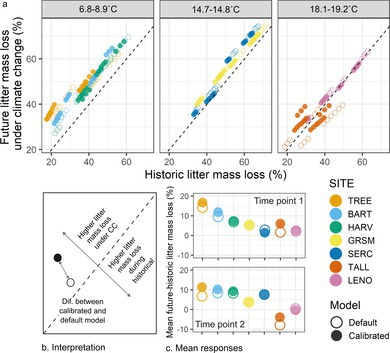

## 1 Introduction

Plant litter decomposition is a critical ecosystem process and globally important carbon (C) flux, which both contributes to soil respiration losses to the atmosphere and serves as a starting point for soil organic matter (SOM) formation. Historically, litter decomposition was thought to be driven predominantly by climate and litter quality, with uncertainty in the relative importance of these two controls (Meentemeyer 1978; Aerts 1997; Cornwell et al. 2008; Zhang et al. 2008; Petraglia et al. 2019). Climate and litter quality controls of decomposition rates have been implemented in process‐based ecosystem models. Typically, abiotic drivers like temperature and moisture availability control rates of mass loss, with litter quality determining litter partitioning into faster and slower decomposing pools (Parton et al. 1987). However, soil and litter microbial communities are also increasingly recognized as important drivers of litter decomposition, with potential implications for responses of litter decomposition to environmental change (Strickland et al. 2009; Allison et al. 2013; Bradford et al. 2021). While some models have recently begun to include functional representations of microbial communities in their formulations (e.g., Wang et al. 2013; Wieder et al. 2014), the use of empirical microbial community data for evaluating these functional representations is still in its infancy. Since ecosystem models are used to predict future ecosystem states under climate change or changes in land management, ensuring they represent our most current understanding of litter decomposition is important for providing confidence in climate‐C cycle feedbacks and management responses.

A persistent question in the field of microbial ecology is how, and to what extent, does a microbial community (defined here to include composition, biomass, and diversity [richness and evenness]) contribute to an ecosystem's function? In the case of litter decomposition, there have been several incubation and field studies supporting the idea that microbial communities have a measurable and distinct effect on litter decomposition rates (Strickland et al. 2009, 2015; Keiser et al. 2011, 2013; Allison et al. 2013; Cleveland et al. 2014; Glassman et al. 2018; Polussa et al. 2021). However, agreement on the importance of the microbial community on litter decomposition is not universal (Smyth et al. 2015; Joly et al. 2023). The influence of the microbial community on ecosystem function has also been explored in process‐based models, which show that including functional representation of microbial communities can improve predictions of SOM or heterotrophic respiration responses to experimental warming (Wieder et al. 2014; Guo et al. 2020). These studies functionally represent microbial communities with different enzyme classes or as copiotrophic and oligotrophic microbes—two examples of how to represent key traits of microbes relevant to ecosystem function in ecosystem models. Copiotroph and oligotroph groupings are a simplistic functional representation but are particularly relevant to microbially explicit soil biogeochemistry models (i.e., models with a microbial biomass pool that affects rates of litter and SOM turnover) because the characteristics of these groups are relatively easily represented with microbial parameters and model structure. Copiotrophs and oligotrophs are characterized as fast‐ and slow‐growing microbes that thrive on nutrient‐rich and ‐poor substrates, respectively (Kuznetsov et al. 1979). As such, the copiotroph‐oligotroph framework offers one axis of microbial functional and physiological diversity with distinct growth rate parameters, preferred substrates, and carbon use efficiencies that can be considered in ecosystem models. Further, these characteristics are also well suited for studying litter decomposition, as substrate quality is an important control on both the relative abundance of these groups and litter decomposition rate (Cornwell et al. 2008; Goldfarb et al. 2011). Importantly, beyond improved predictions, models with functional representations of microbial communities also improve ecological realism by including a driver that has been shown to be important for ecosystem functions. Further, using empirical microbial community data for parameterizing these models has reduced parameter uncertainty (Guo et al. 2020; Tao et al. 2024). However, we know of no modeling study that has attempted to integrate empirical microbial community data to evaluate controls of leaf litter decomposition, despite numerous empirical studies indicating its potential importance.

Because we use process‐based models as tools to assess future responses to climate change, it is important they correctly represent and balance empirically important drivers and controls, thus providing the proper framework for projecting responses to future climate change (Sulman et al. 2014). Models that implicitly represent microbial activity are able to predict leaf litter decomposition rates well when compared to field experiments (Bonan et al. 2013). However, while some models might be able to predict current conditions accurately, that does not necessarily translate to *confidence* in their future predictions. To ensure confidence in future projections, model structures should represent current empirical understanding and include parameterizations informed by empirical data (Bradford et al. 2016; Butler et al. 2021; Le Noë et al. 2023). Building this confidence is particularly important in the context of climate change, for which we have no direct empirical analog. Other recent studies have used empirical datasets to calibrate microbially explicit soil biogeochemical models for prediction of leaf litter decomposition datasets, but neither of these incorporate microbial community data in their calibrations, providing a gap in model evaluation of this empirically important driver (Aas et al. 2024; Juice et al. 2024). Importantly, by using model calibration to improve representation of drivers of litter decomposition, rather than solely traditional goodness‐of‐fit metrics, we can improve model confidence by better including mechanistic representation.

We calibrated a microbially‐explicit soil biogeochemistry model to represent drivers of leaf litter decomposition (referred to as “litter decomposition” from here forward), specifically including microbial community data, represented as the copiotroph to oligotroph ratio, in our calibration. This work is timely given the demonstrated importance of microbial communities for mediating rates of litter mass loss, process‐based model capacity for representing those communities, and the broader importance of representing observational drivers in process‐based models. We use a unique and comprehensive empirical dataset, which includes variation in soil moisture, litter lignin:N ratios, and bacterial copiotroph:oligotroph ratios (representing climate, litter quality, and microbial community, respectively) at both local and regional scales, to assess drivers of litter decomposition. Other important drivers of litter decomposition, such as soil nutrient availability (which may or may not be tied to litter quality) and fauna (Hobbie 2015; García‐Palacios et al. 2013), are not addressed in our work and associated limitations are discussed in Section 4.3. We use a novel Monte Carlo parameterization method to calibrate the model to both litter decomposition rates and empirical drivers of litter decomposition. We compare this calibrated model with empirically‐informed parameters to a model with parameters not informed by the empirical litter decomposition data. Because the calibrated model uses empirically‐informed parameters and parameters control model dynamics, we hypothesized that the calibrated model would alter the dynamics of litter decomposition with implications for ecological processes, particularly with respect to the microbial community. Specifically, we expected modelled representations of lignin:N and soil moisture effects on litter decomposition to be similar to empirical data before calibration as negative and positive effects, respectively. In contrast, we expected empirically‐informed model dynamics to show that copiotrophs would be the dominant decomposers of litter, due to their faster growth rate, and that microbial processes represented in MIMICS such as catabolic capacity, carbon use efficiency, and turnover are influential on litter decomposition. We also hypothesized that calibration would increase responses of decomposition to climate change because of the expected dominance of copiotrophs which have faster growth rates and hence, decomposition.

## 2 Materials and Methods

Two key limitations to integrating soil microbial data into ecosystem‐scale process‐based models are (1) that empirical microbial data are rarely able to be plugged directly in for model parameters and (2) that calibrating to absolute values of microbial pools does not necessarily cause the model to have the correct underlying drivers. To address these issues, we calibrate our model to empirical effect size estimates (e.g., coefficients from linear mixed‐effects models) for proxies of three key drivers of litter decomposition: soil moisture, litter lignin:N ratio, and copiotroph:oligotroph ratio. To that end, we first describe the empirical data and how we calculate empirical effect sizes (Section 2.1). Section 2.2 then describes how we set up our model to provide comparable modelled effect sizes. Next, we describe how we carried out our calibration to choose parameters that most closely align empirical and modelled effect sizes, as well as empirical decomposition rates, and subsequently validate that calibration (Section 2.3). Finally, Section 2.4 describes how we evaluated predictions of litter decomposition under climate change from our calibration in comparison to the initial parameters.

### 2.1 Empirical Data Collection and Analysis

We conducted a highly replicated litterbag study at 10 temperate forest U.S. National Ecological Observatory Network (NEON) sites (Table 1). Seven sites, where we measured microbial data, were used for model calibration and the remaining three sites, where we did not have microbial data, were used for model validation. At each site, 12 plots (1 × 1 m^2^) were set out for each of the three dominant tree species, resulting in 36 plots per site. At each plot, ~5 g of leaf litter from an adjacent canopy tree was collected at peak leaf fall and encased in litterbags (20 × 20 cm). For this study, we used 2 litterbags per plot (for each of the two collection time points), 72 per site, and 720 total litterbags for all 10 sites. Bags had an underside mesh with a 54 μm aperture to prevent loss of litter as it became fragmented, and a topside mesh with an aperture width of 1.84 mm to permit mesofauna access. Plots were arrayed in a closed loop along the periphery of the airshed of the NEON eddy covariance tower (spanning 5–88 ha depending on site) to capture within‐site heterogeneity. Plots were established in Fall 2021 and after approximately 10 and 21 months (time points 1 and 2, respectively), litterbags were collected, stored at 4°C for up to 1 week, cleaned with a fine brush and tweezers to remove any soil and non‐litter debris, and weighed to record total fresh mass remaining. Litter mass loss was determined as the percent of initial mass remaining. Because we had C concentration data from only initial litter, percent initial mass remaining was assumed to be equivalent to percent C remaining.

**TABLE 1:** Site data used to force MIcrobial‐MIneral Carbon Stabilization (MIMICS) model in this study ordered as the sites used for calibration or validation (indicated by the “(val.)” following their site names), and then from coldest to warmest. Sites used for calibration from the U.S. National Ecological Observatory Network (NEON) include Treehaven (TREE), Bartlett Forest (BART), Harvard Forest (HARV), Great Smoky Mountains (GRSM), Smithsonian Environmental Research Center (SERC), Talladega (TALL), and Lenoir Landing (LENO). Sites used for validation from NEON include University of Notre Dame Environmental Research Center (UNDE), Mountain Lake Biological Station (MLBS), and Smithsonian Conservation Biology Institute (SCBI).

Site (state)	Annual litterfall (gC m^−2^ year^−1^)	Mean soil temperature (°C)	Mean soil moisture scalar	Max soil moisture multiplier	Min soil moisture multiplier	Clay (%)	LIG:N 1	LIG:N 2	LIG:N 3
TREE (WI)	519	6.8 (10.8)	0.44 (0.27)	1.10	0.90	5	12	30	32
BART (NH)	613	8.5 (9.3)	0.55 (0.29)	1.07	0.93	12	15	34	40
HARV (MA)	637	8.9 (9.1)	0.66 (0.25)	1.06	0.94	16	27	33	65
GRSM (TN)	853	14.7 (6.5)	0.80 (0.06)	1.06	0.94	16	21	25	43
SERC (MD)	744	14.8 (8.0)	0.62 (0.09)	1.03	0.97	12	15	20	33
TALL (AL)	608	18.1 (6.3)	0.66 (0.11)	1.08	0.92	3	25	37	112
LENO (AL)	841	19.1 (6.1)	0.71 (0.12)	1.03	0.97	21	32	35	54
UNDE (MI; val.)	591	6.1 (10.3)	0.47 (0.32)	1.07	0.93	6	13	31	34
MLBS (VA; val.)	639	9.5 (7.5)	0.70 (0.19)	1.03	0.97	11	6	26	37
SCBI (VA; val.)	640	13.0 (8.1)	0.61 (0.11)	1.06	0.94	32	13	17	34

*Note:* Community Land Model (CLM) output was used to determine litterfall, temporally‐averaged soil temperature (with standard deviation), and a temporally‐averaged soil moisture scalar (with standard deviation), a 0–1 scalar calculated from soil water potential in CLM. Clay content is from NEON megapit measurements interpolated to CLM depths; we use the clay content at 1 cm depth. The soil moisture scalar (which is unitless) is multiplied by the max and min soil moisture multipliers to represent moisture variability within a site as informed by field data. Lignin‐to‐nitrogen (LIG:N) values were averaged from field collected litter for three tree species from each site. These are ordered in ascending order (e.g., decreasing litter quality; LIG:N 1–3).

To determine the relative importance of climate, litter quality, and microbial community for litter decomposition, we used the proxies of soil moisture, litter lignin:N, and bacterial copiotroph:oligotroph ratio, respectively, at the seven sites where microbial data were collected (i.e., not validation sites; Table 1). Soil moisture was measured at experiment initialization and each collection time point in soil under each litterbag (108 measurements per site) with a time domain reflectometry (TDR) probe, inserted at a 45° angle to ~5 cm depth (Hydrosense II, Campbell Scientific, Logan, UT, USA). For initial litter, lignin was determined using an acid digestion (AOAC Official Method 973.18 1997) and percent N was measured on dried, ground litter using a Thermo EA IsoLink CN connected to a Thermo isotope ratio mass spectrometer (Thermo, Bremen, Germany). Lignin:N was calculated as the mean lignin of 1–2 laboratory replicates of leaf litter of each tree species within each site (*n* = 3 per site) over plot‐level percent N of litter (e.g., for each replicate of each species within a site; *n* = 36 per site).

We leveraged 16S rRNA gene amplicon sequence data from soil samples at experiment initiation (temporally matching the litter lignin and N measurements) in 5–12 plots (depending on sampling extent and data quality) at each of the seven sites to obtain bacterial copiotroph:oligotroph ratios to be used in our statistical model (Polussa and Oliverio 2025). Five plots were used at GRSM and HARV, 8 at BART, 10 at TREE, 11 at LENO and TALL, and 12 at SERC. We use copiotroph and oligotroph groupings to represent the microbial community because these groups are represented in the process‐based model used in this study but acknowledge there are multiple ways to represent functional traits of microbial communities. In brief, DNA was extracted from 200 to 700 mg soil using the Zymo Quick‐DNA Fecal/Soil Microbe DNA Miniprep Kit and then amplified using a 250‐bp fragment of the V4–V5 region of the 16S rRNA gene. Sample concentrations were normalized and sequenced on the Illumina MiSeq platform with 2 × 150‐bp paired‐end chemistry at the University of Colorado Next Generation Sequencing Facility along with negative controls to check for possible contamination. We processed the raw reads to amplicon sequence variants (ASVs) with the DADA2 pipeline (Callahan et al. 2016) as per Shepherd and Oliverio (2024). Samples were rarefied to 2447 reads per sample and bacterial copiotroph: oligotroph was calculated as the sum of copiotroph ASV counts over the sum of oligotroph ASV counts per plot. Bacterial taxa were classified as copiotrophs or oligotrophs using phylogeny, which has been shown to be predictive of growth rate, the main trait distinguishing copiotrophs and oligotrophs (Walkup et al. 2023). Classification was across multiple taxonomic levels, ranging from phylum to genus, using publicly available criteria established in Averill et al. (2021) and Ho et al. (2017). Criteria were based on a literature review prioritizing functional studies with pure culture work, stable isotope probing, and coupled community and physiological analyses. There were 13 genus‐level, 2 class‐level, 4 order‐level, 4 family‐level, and 13 phylum‐level classifications, with preference given to finer classifications. Copiotrophs generally included taxa within Alphaproteobacteria, Bacteroidetes, and Gammaproteobacteria, while oligotrophs were represented by Acidobacteria, Planctomycetes, and Verrucomicrobia.

Here, we note some limitations associated with our microbial data and how these were addressed, if possible. We use soil bacterial communities rather than those in litter with the assumption that the community in the surface soil is an adequate representation of decomposer community variation across experimental sites. Future investigation of the colonization and succession of leaf litter decomposing communities would be valuable. However, here we use data on soil bacterial communities collected when litterbags were first placed in the field given that Barbour et al. (2022) found that the majority of taxa overlap between soil and litter and using initial soil microbial community as a proxy for microbial variation is common practice (e.g., Strickland et al. 2009; Keiser et al. 2011). Fungal decomposers are key drivers of litter decomposition, but we do not consider them here due to a lack of phylogenetic data that is tied to growth rate for soil fungi. Alternative classifications and recent work characterizing fungal life history traits offer promise for future work (also addressed in Section 4.3; Crowther et al. 2014; Camenzind et al. 2024; Leifheit et al. 2024). The bacterial relative abundance data we use (e.g., from 16S) does not directly translate to model output, which is the C content of the copiotroph and oligotroph pools. Because the model does not discern between fungi and bacteria and instead represents copiotrophic and oligotrophic strategies more generally, this is another limitation to not having a representation of empirical fungal data. However, by using the ratio of copiotrophs to oligotrophs as our predictor, we aim to compare the relative dominance of copiotrophs vs. oligotrophs rather than compare absolute amounts of either group, with the assumption that dominance of a given bacterial strategy might be indicative of a comparable fungal strategy (Ma et al. 2023). Finally, in our classification process, anywhere from 6% to 70% of taxa in a sample are not classified as copiotrophs nor oligotrophs; this lack of classification was similar across sites (Figure S1a). To assess the influence of this, we performed a sensitivity analysis where we designated non‐assigned taxa as either copiotrophs or oligotrophs (100% copiotrophs and 0% oligotrophs, 90% copiotrophs and 10% oligotrophs, 80% copiotrophs and 20% oligotrophs and so on) and found the largest effect of these unassigned values was to increase the effect size of copiotrophs: oligotrophs by approximately 10 units (Figure S1b,c), so we included this observational uncertainty when calibrating our model (described in Section 2.3). We did not further consider unassigned taxa for our bacterial copiotroph: oligotroph estimates. Additional uncertainty associated with the classification of these groups is due to classification being less successful at coarser taxonomic resolution (e.g., phyla; Ho et al. 2017; Stone et al. 2023; Walkup et al. 2023). To reduce this uncertainty, the method of classification we chose (Ho et al. 2017; Averill et al. 2021) prioritized finer resolution taxonomy when available and was based on data using the best available methodology (e.g., pure culture work, stable isotope labelling approaches, or community analyses supported by physiological characteristics; Ho et al. 2017). To account for this uncertainty in our calibration, we weigh matching litter decomposition rates more highly than matching the empirical driver of bacterial copiotroph: oligotroph ratio when choosing best parameter sets (described in Section 2.3).

To determine the relative importance of different drivers of litter decomposition, we created a linear mixed effects model of the field data with soil moisture, lignin:N, and bacterial copiotroph:oligotroph ratios as drivers and percent litter mass loss at time points 1 and 2 as the response variables. Plot nested within site was a random effect in the model to account for potential temporal correlation from sampling litterbags from the same plots, and site reflected the spatial non‐independence of multiple plots within a forest site. Decomposition kinetics in MIMICS are temperature sensitive and have been calibrated to site‐level variation in temperature with data from the Long‐term Inter‐site Decomposition Experiment Team (LIDET) study (Harmon et al. 2009). Given our lack of plot‐level soil temperature measurements, which would supplement the site‐level calibration and were not collected because of minimal within‐site spatial variation (e.g., 10× less variable than soil moisture; Loescher et al. 2014), we do not include temperature in our within‐site scale model calibration efforts. However, temperature is a notable control on litter decomposition (e.g., Petraglia et al. 2019) and so we assessed the influence of including site‐level soil temperature (Table 1) for our empirical effect size estimates. We found temperature had relatively little influence on the model R^2^ and reduced the relative effect sizes comparably across the other drivers, minimally changing their direction and magnitude relative to one another (Figure S2). However, temperature still had a considerable effect size, and we discuss the importance of future studies including temperature in Section 4.3. For the linear mixed effects model, soil moisture was represented as the mean volumetric water content taken over three timepoints (0, 10, and 21 months) at each plot within each site. Soil moisture was log transformed to meet linearity assumptions, and all three independent variables were standardized to increase comparability (i.e., unstandardized coefficient values reflect different unit scales). We limit our empirical statistical analysis to plots with 16S data, where each plot has a unique soil moisture and lignin:N measurement (*n* = 124 observations over two time points; dataset available at Rocci, Pierson, and Wieder 2024).

### 2.2 Model Setup

We use the MIcrobial‐MIneral Carbon Stabilization (MIMICS) model (Figure S3; Wieder, Grandy, et al. 2015; Rocci, Pierson, and Wieder 2024) to project rates of leaf litter decomposition at each of the seven NEON sites. MIMICS is well‐suited for the goals of our study because it is one of relatively few ecosystem‐scale process‐based models to include a functional representation of microbial communities, which we can couple to our empirical data to evaluate the influence of microbial community composition on litter decomposition rates. A detailed description of MIMICS can be found in Wieder, Grandy, et al. (2015) but briefly, MIMICS separates litter into a metabolic and structural litter pool (LITm and LITs) based on litter quality (as a linear function of lignin:N content of litterfall). In the model, LITm is preferentially decomposed by a copiotrophic microbial group (MICr) and LITs by an oligotrophic microbial group (MICk). Microbial turnover subsequently contributes C to physically protected and chemically protected soil organic matter pools (SOMp and SOMc), as well as to the available SOM pool (SOMa), from which microbes can also assimilate C (Figure S3). We calculated steady‐state pools in MIMICS using site‐specific inputs (described below) using a standard ordinary differential equation solver from the RootSolve package (Soetaert and Herman 2009). As in Wieder, Grandy, et al. (2015), we subsequently added litterbag metabolic and structural litter pools that decomposed but did not influence microbial biomass or SOM pools in the underlying model. In other words, all decomposed litter from the litterbag was immediately lost from the system with the assumption that the amount of C in the litterbag was much smaller than and would have minimal effect on the steady‐state C pools in the model.

In our model experiments at each of the NEON sites, we simulated within‐site heterogeneity by decomposing three litter types (Table 1) across a gradient in soil moisture (described below). Inputs for MIMICS (e.g., litterfall, soil temperature, soil moisture, and percent clay; Table 1) were generated from site‐scale simulations using the Community Land Model version 5.2 (CLM5.2) forced with meteorological observations (2018–2022) from the National Ecological Observatory Network (NEON) data version 3, as described in Lombardozzi et al. (2023). For the LENO site, input data were only available for 2021–2022 and for the HARV site for 2018–2021. From these NEON‐CLM data, annual means or sums of input data for each site were used to calculate steady state. Daily input climatologies (e.g., averaged across years) were used to force litterbag simulations. Litterbag simulations ran for 3 calendar years, where litterbags were added to the model on November 11th (the average day of litterbag deployment across sites) and decomposed for the remaining ~2 years. We corrected anomalously high litterfall data simulated by CLM at the TALL site (verified using data from Jevon et al. 2022) by using an empirical relationship between modeled annual litterfall at the other sites and GPP estimates from NEON flux tower measurements averaged over 2018–2021 (NEON 2023; Figure S4).

To represent the variability in soil moisture in each site, we calculated the mean and lower and upper 95% confidence intervals of observed field soil moisture within each site and then determined a minimum and maximum soil moisture multiplier for each site by dividing the lower and upper 95% confidence intervals by the mean at each site. We then multiplied the soil moisture scalar from both annual and daily CLM output (a 0–1 scalar on decomposition rate calculated from soil water potential in CLM) by minimum and maximum soil moisture multipliers, in addition to having the soil moisture scalar from CLM output (3 soil moistures per site; Table 1). This allowed us to represent soil moisture variability while minimizing the necessary number of unique model runs, thereby allowing us to run a large number of parameter sets in the Monte Carlo parameterization, described in Section 2.3.

For our initial model setup, we used MIMICS parameters from Wieder, Grandy, et al. (2015) with two main modifications. First, we replaced the previous density dependent parameterization in MIMICS with the beta parameterization of density dependent microbial turnover (Equation 1) described by Georgiou et al. (2017), such that higher microbial biomass or higher betas cause more microbial turnover:

(1) $$\text{MICtrn}={\mathrm{MIC}}^{\text{beta}}\times \mathrm{tau}\times \mathrm{fS}$$

where MICtrn is microbial turnover, MIC is the microbial biomass carbon pool, beta is the parameter from Georgiou et al. (2017), tau is the ratio of biomass that is turned over, and fS is the fraction of turnover allocated to a given SOM pool. Implementing beta reduced the oscillatory behavior of microbial biomass in our preliminary simulations with MIMICS. Second, we reduce the microbial catabolic capacity (*V*_max_) by 40% to create initial litter decomposition kinetics that agree with our field data. We reduce microbial catabolic capacity to ensure we are not correcting a kinetics bias with our calibration, whereas the goal of our calibration is to better represent drivers of litter decomposition. The initial MIMICS parameters (e.g., from Wieder, Grandy, et al. 2015) combined with the use of beta and reduction of *V*_max_ represent the default model parameters that are the starting point for our calibration (Table S1). For historical simulations (e.g., using forcing data climatology from 2018 to 2022, which we expect to be similar to our field experiment period), we ran 63 unique model runs (7 sites × 3 litters × 3 soil moistures). These historical runs with the default model parameters are referred to as the “default” model moving forward.

To determine the effects of soil moisture, litter lignin:N ratio, and copiotroph:oligotroph ratio on historical modeled litter mass loss, we created a similar statistical model as for the observational data. From here forward, we use copiotroph:oligotroph ratio rather than bacterial copiotroph:oligotroph ratio to represent both the empirical and model estimates, the former of which is based solely on bacteria. From model output, we calculated litter mass loss for the two site‐specific field collection time points (on average, 10 and 21 months) and extracted initial copiotroph:oligotroph ratios (e.g., MICr:MICk at steady state), the temporally‐averaged soil moisture scalar for each of the three soil moistures, and the litter lignin:N ratio. We then use the latter three variables in a linear mixed effects model predicting litter mass loss to estimate the effect sizes of climate (soil moisture), litter quality (lignin:N), and microbial community (copiotroph:oligotroph) with “plot” (e.g., each litter type and soil moisture combination) nested within site as a random variable. As with the observational data, we log transformed soil moisture to meet linear model assumptions and standardized all predictors to make their effect sizes more comparable. We checked the statistical models for observational data and default and calibrated model output (described in Section 2.3) for collinearity and found that predictors in each model had variance inflation factors less than 5, suggesting correlations between the variables were not severe and our statistical models were appropriate.

### 2.3 Calibration and Validation

For the calibration, we start from the default model with the parameter modifications described in Section 2.2. To calibrate the model to empirically important drivers of litter decomposition, we first conducted a one‐at‐a‐time sensitivity analysis to identify the parameters that had the largest effect on litter decomposition rates and drivers that were simulated by MIMICS. This identified two parameters related to microbial turnover and two related to catabolic capacity (Table 2) that were used in a Monte Carlo calibration following the approach by Pierson et al. (2022). The combination of parameters was assessed by dropping one parameter at a time to ensure each parameter was required to meet calibration goals. Model calibration was performed using 5000 random parameter sets of our selected four parameters, with descriptions and ranges summarized in Table 2. We ensured that we had a sufficient number of random model runs by running a sensitivity test to ensure similar parameter sets were retrieved if we left out 10% of the runs (Figure S5). As above, we calculated modelled litter mass loss for each site, litter type, and soil moisture variant at each timepoint.

**TABLE 2:** Descriptions, default values, multiplier ranges, and calibrated multipliers values for the MIMICS parameters used in the calibration. The three best (see methods for explanation of “best”) sets of calibrated multipliers are shown in sub‐columns under the “Calibrated parameter multipliers” column (e.g., Set 1, Set 2 and Set 3).

Parameter name	Description	Default value	Multiplier range	Calibrated parameter multipliers		
				Set 1	Set 2	Set 3
tau_r	The litter quality multiplier on copiotrophic turnover	0.3	0.3–2	1.33	0.58	0.82
beta	A multiplier for density dependent microbial turnover	1.5	0.67–1.33	0.76	0.72	0.74
vMODm	Overall multiplier on maximum metabolic litter decomposition rate	10, 3^a^	0.5–2	1.76	1.62	1.57
vMODs	Overall multiplier on maximum structural litter decomposition rate	2, 3^a^	0.5–2	0.93	0.92	0.95

^a^ For copiotrophic and oligotrophic microbes, respectively—note the same multiplier is applied for each microbial group.

After running all simulations, we filtered the 5000 parameter combinations based on the following four criteria. Parameter sets were kept if they: (1) produced litter mass loss rates that fell within observed minimum and maximums for each site at each timepoint, (2) produced relative effect sizes within 10 units of observed relative effect sizes for soil moisture, litter lignin:N, and copiotroph:oligotroph ratio, (3) produced root mean squared errors (RMSEs) less than 5.4% (chosen to ensure multiple good‐fitting parameter sets) for observed versus modeled litter mass loss, and (4) maintained viable copiotrophic *or* oligotrophic microbial groups. This filtering provided three best parameter sets that we use in the “calibrated” model (Table 2). We prioritized more closely matching litter decomposition over effect sizes since we have more certainty in empirical litter mass loss estimates than empirical effect size estimates, particularly for the copiotroph:oligotroph ratio.

Independent validation of the calibrated parameter sets was conducted using observed field decomposition rates from NEON forest sites that were not used in the aforementioned calibration activities. Validation sites were three experimental field sites that applied the same experimental design for litterbag experiments, but where no 16S data were collected and so they could not be used for calibration (Table 1). We ran MIMICS simulations for validation sites with inputs generated from CLM (as was done for sites used in model calibration). At validation sites, we ran simulations with both default and calibrated parameters and quantified litter mass loss compared to field observations. From our calibrated model results, we compared the steady state pool sizes, litterbag decomposition rates, and the copiotroph:oligotroph ratio to evaluate the differences between the calibrated and default models.

### 2.4 Implications Under Climate Change

To evaluate the influence of the new calibration on litter mass loss under climate change, we ran both the default and the calibrated MIMICS model under a high climate change scenario, Shared Socioeconomic Pathway (SSP) 3–7.0, through the year 2100. Briefly, the anomaly forcing provides a smooth transition from observed to future climate that is generated by subtracting the climatological mean baseline atmospheric state (2005–2014) from the mean monthly atmospheric states that were simulated from a Community Earth System Model version 2 (CESM2) SSP3‐7.0 simulation (Wieder, Cleveland, et al. 2015; Danabasoglu et al. 2020; Jay et al. 2023). We then added these anomalies to historical climate data cycled over the observational record (2018–2022) for each NEON site and used these data as MIMICS inputs (e.g., litterfall, soil temperature, and soil moisture). To compare to our historical runs, we used input data for the three‐year period from 2072 to 2074, 50 years after the historical data end date of 2022, to force the default and calibrated models. We then compared decomposition rates for simulations of both the default and calibrated models (including the three top parameter sets) from the future run to the historical run to determine the effect of climate change for both models. We note that using other future time periods did not strongly alter the interpretation of the results besides decomposition becoming faster as climate change progressed.

To evaluate the differences between the calibrated and default model responses to climate change, we built a linear mixed effects model with the percent difference in the calibrated versus default climate change responses for each time point, litter quality, and soil moisture combination for each site (*n* = 7 sites × 2 time points × 3 litter qualities × 3 soil moistures = 126) as the response variable. As the driving variables, we used site clay, site soil moisture variability, and “plot” litter quality with either historical annual litterfall, mean soil temperature, and “plot” soil moisture, the change in those variables under climate change, or the future values of those variables. As with the above linear mixed effect models, “plot” (e.g., each litter quality, soil moisture, site combination) nested within site was the random effect and soil moisture was log transformed to meet the assumptions of the linear model. All statistical and model analyses were carried out in R statistical software (R Core Team 2024).

## 3 Results

### 3.1 Empirical Data and Model Calibration

**FIGURE 1:**
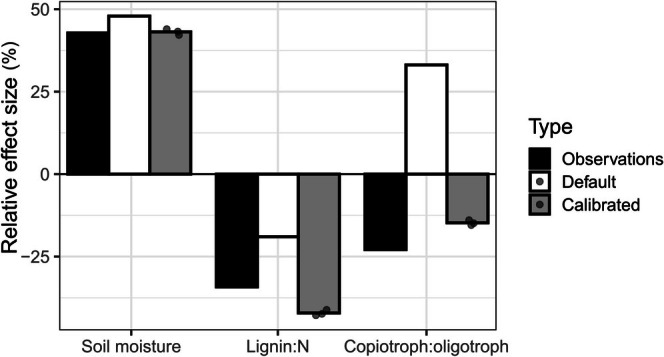
Relative effect sizes for linear mixed effects models (e.g., model coefficients relativized to 100%) predicting litter mass loss using soil moisture (volumetric water content and soil moisture scalar for observations and model output, respectively), litter quality (lignin:N), and microbial community (copiotroph:oligotroph) as independent variables for the observations, default model, and calibrated model (fill color). Points show effect sizes for each parameter set in the calibrated model.

**FIGURE 2:**
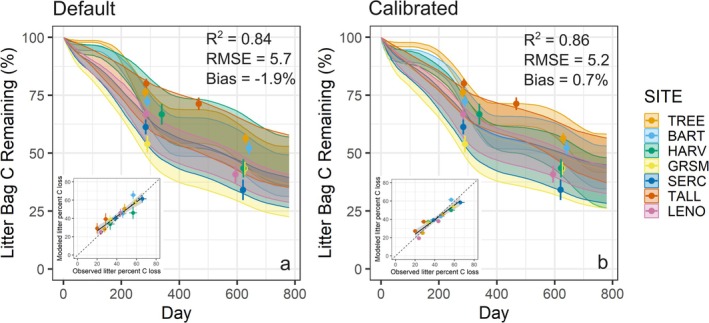
Litter mass remaining in (a) default and (b) calibrated models compared to observations. Shading shows minimum and maximum decomposition predictions from the model, including variability due to three litter types and three soil moistures, and for the calibrated model, three parameter sets (*n* = 9 and *n* = 27 simulations per site for default and calibrated results, respectively). Points with 95% confidence intervals show variability in observed litterbag mass loss at collection time points 1 and 2. Insets show comparison of modeled and observed decomposition, with dashed line depicting the 1:1 line. Sites are ordered from coldest (TREE) to warmest (LENO).

The observed field litterbag data revealed that soil moisture was positively related, and lignin:N and copiotroph:oligotroph were negatively related to rates of litter mass loss (Figure 1; Figure S6). The default MIMICS model was able to reproduce observed rates of litter mass loss over the two observational time points (Figure 2a) but had considerably different effect size estimates than the observations, particularly for the copiotroph:oligotroph ratio, which was in the opposite direction from the observations (Figure 1). The calibrated model, for which we average over three simulations with the best parameter sets, was also able to reproduce litter mass loss over the two observational time points (Figure 2b) and was better aligned with the effect sizes from the observational data (Figure 1).

### 3.2 Validation

**FIGURE 3:**
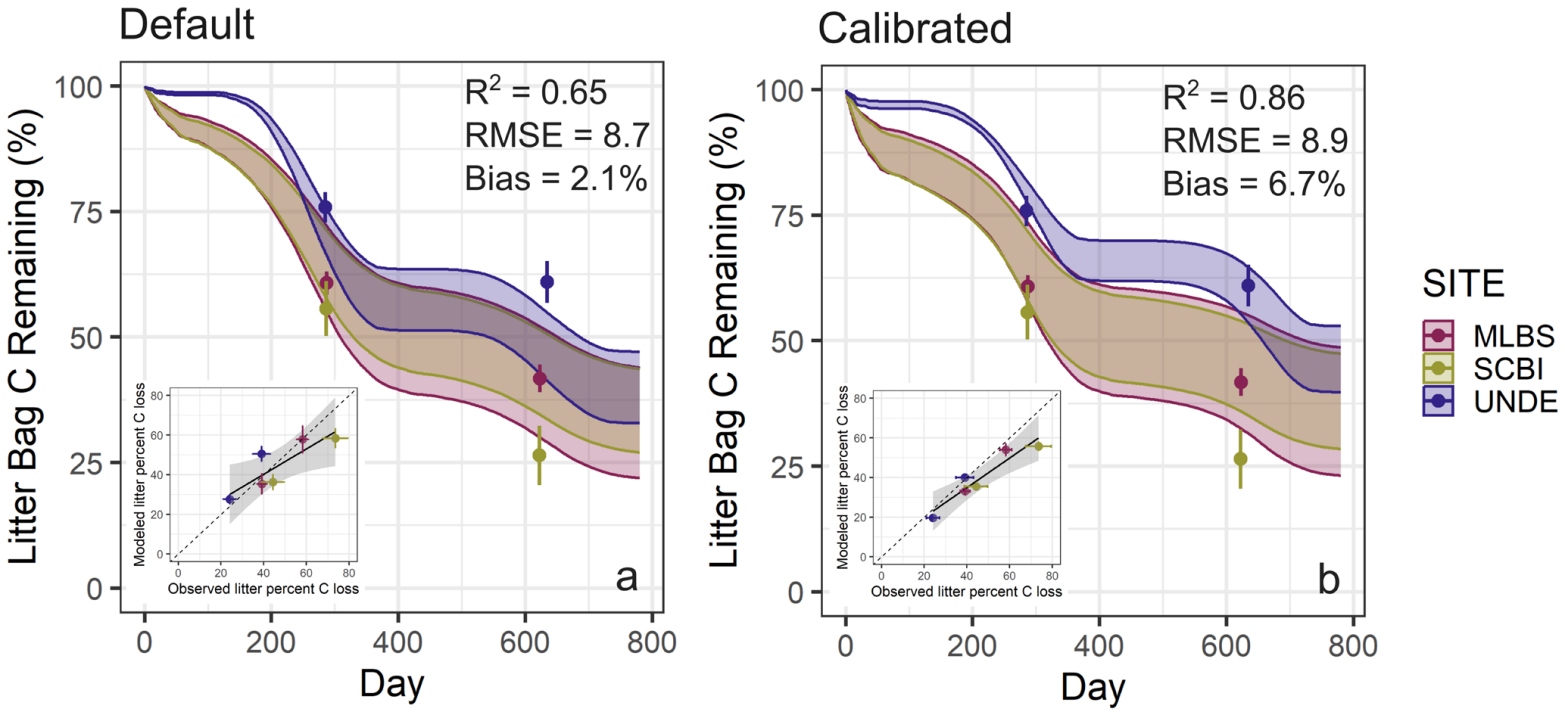
Performance of the (a) default and (b) calibrated models for the validation sites. Shading shows minimum and maximum decomposition predictions from the model, including variability due to three litter types and three soil moistures, and for the calibrated model, three parameter sets (*n* = 9 and *n* = 27 simulations per site for default and calibrated results, respectively). Points with 95% confidence intervals show variability in observed decomposition. Insets show comparison of modeled and observed decomposition with dashed line depicting the 1:1 line.

We independently validated results with simulations at three additional NEON sites (not used in the calibration) and showed that parameter sets calibrated at the seven sites with microbial data similarly improve simulated rates of litter decomposition. Specifically, the calibrated parameter sets provided model simulations that were more similar to observations, especially at UNDE, but had higher bias compared to the default parameter set (Figure 3). Effect sizes for the default model in the validation runs were in similar directions as the historical runs, albeit slightly different magnitudes (e.g., stronger and weaker effects of soil moisture and litter quality, respectively; Table 3). However, effect sizes for the calibrated model in the validation runs were quite different from the historical runs (particularly for litter lignin:N ratio and copiotroph:oligotroph ratio) and more variable among parameter sets (Table 3).

**TABLE 3:** Relative effect sizes (%) from linear mixed effect models predicting litter mass loss in the validation sites and under climate change for the default and calibrated model, with historical runs added for comparison. The independent variables in the model are soil moisture (soil moisture scalar), lignin:N, and copiotroph:oligotroph. Ranges for calibrated models show the range across the three best parameter sets.

Run type	Model type	Soil moisture	Lignin:N	Copiotroph:oligotroph
Historical	Default	47.9	−19.0	33.1
Historical	Calibrated	42.2 to 44.0	−41.1 to −42.8	−14.0 to −15.5
Validation	Default	23.7	−45.2	31.0
Validation	Calibrated	31.0 to 51.6	−8.3 to 11.7	40.1 to 57.2
Climate change	Default	38.0	−11.9	50.0
Climate change	Calibrated	49.7 to 52.9	−39.0 to −44.3	6.1 to 8.1

### 3.3 Comparison of Default and Calibrated Models

**FIGURE 4:**
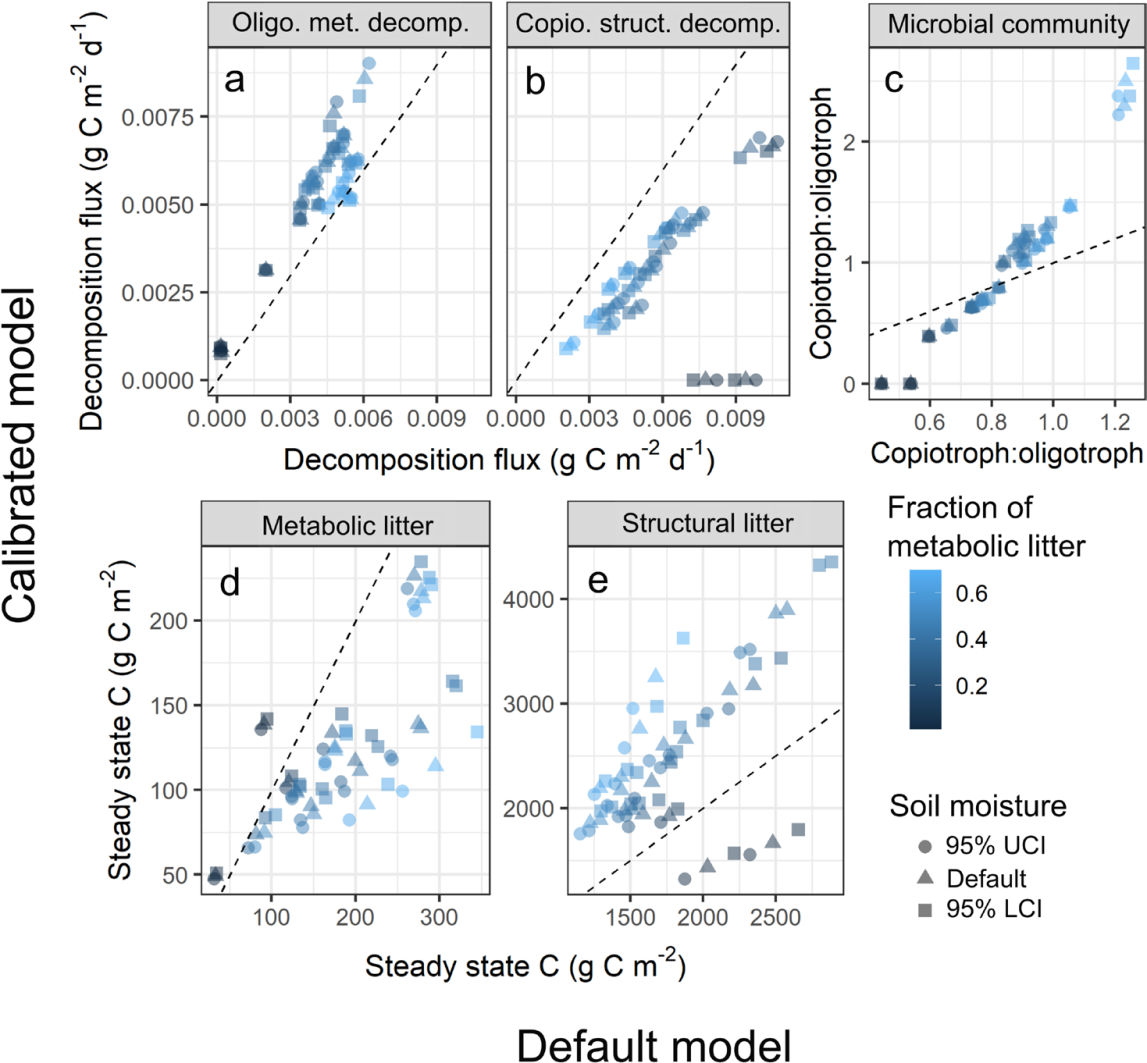
Comparison of calibrated model output (*y*‐axes; mean of top 3 parameter sets) vs. default model output (*x*‐axes) for (a) decomposition rates of metabolic litterbag litter by oligotrophs and (b) structural litterbag litter by copiotrophs. Plots a and b are labeled as the “decomposition flux” because these values are the flux rate between respective litter and microbial pools in the model. Comparison is also shown for (c) copiotroph:oligotroph ratios and steady state C pools in (d) metabolic and (e) structural litter in the default and calibrated models. Plots a and b are model output averaged over the litterbag period and plots c–e depict the modelled steady state pool. Dashed line depicts the 1:1 line. Points are colored by litter quality and shapes show variation in soil moisture.

By changing parameter values to better align effect sizes in MIMICS with those from the observational data, we also modified the dynamics of MIMICS. Averaging results from the best parameter sets in the calibrated model, we find our calibrated results shift decomposition rates; oligotrophs decompose metabolic litter more rapidly and copiotrophs decompose structural litter more slowly, compared to the default parameterization (Figure 4a,b). At the same time, litter quality more strongly shapes microbial communities in the calibrated model (Figure 4c) but the strength of this effect varies across parameter sets (Figure S7). These effects in the calibrated model are associated with less metabolic litter and more structural litter at steady state compared to the default model, as oligotrophs decompose more metabolic litter and copiotrophs decompose less structural litter (Figure 4d,e). Notably, in all three calibrated parameter sets, coexistence of copiotrophic and oligotrophic microbes is not maintained at low litter quality. Specifically, in the calibrated results, only oligotrophs survived with eastern white and longleaf pine ( *Pinus strobus* and *Pinus palustris* ) litter (lignin:N > 60) at HARV and TALL, respectively. Differences in the three best parameter sets chosen through calibration could indicate equifinality (multiple parameter sets working equally as well for modelling targets) and/or provide ecological insight (Supporting Information: Text A; Figure S8).

### 3.4 Decomposition Response to Climate Change

**FIGURE 5:**
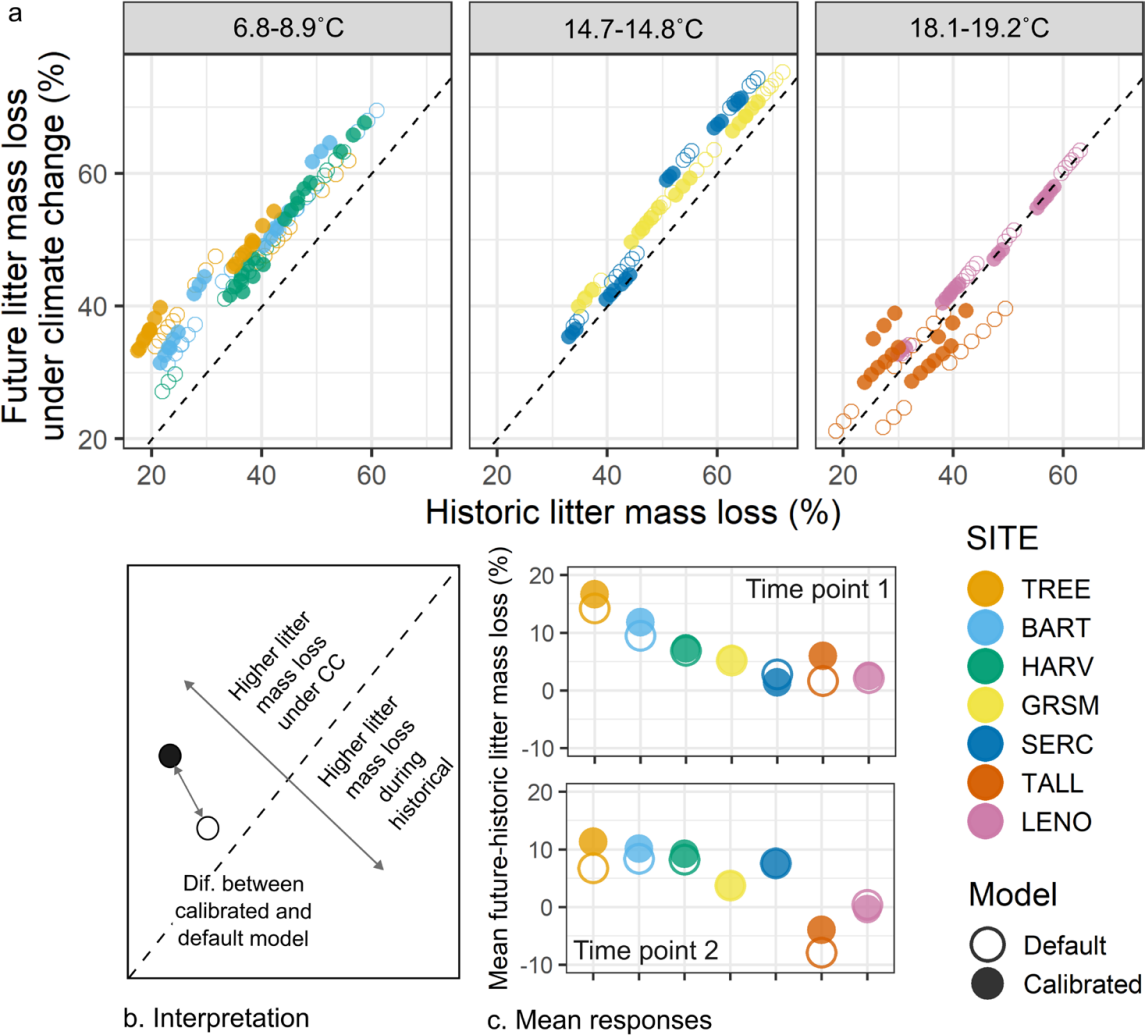
Responses of litter mass loss in default (open) and calibrated (filled) model to 50 years of climate change under the SSP 3–7.0 scenario. The dashed lines in (a) and (b) represent the 1:1 line. Calibrated points are means of the three top parameter sets. Facets in (a) group sites by their mean soil temperatures over the historic period. Plot (b) shows the interpretation of plots in (a) and plot (c) shows the mean change in litter mass loss under climate change for each site (color) and for the default (open) and calibrated (filled) models for time points 1 and 2. Sites are ordered from coldest (TREE) to warmest (LENO).

**FIGURE 6:**
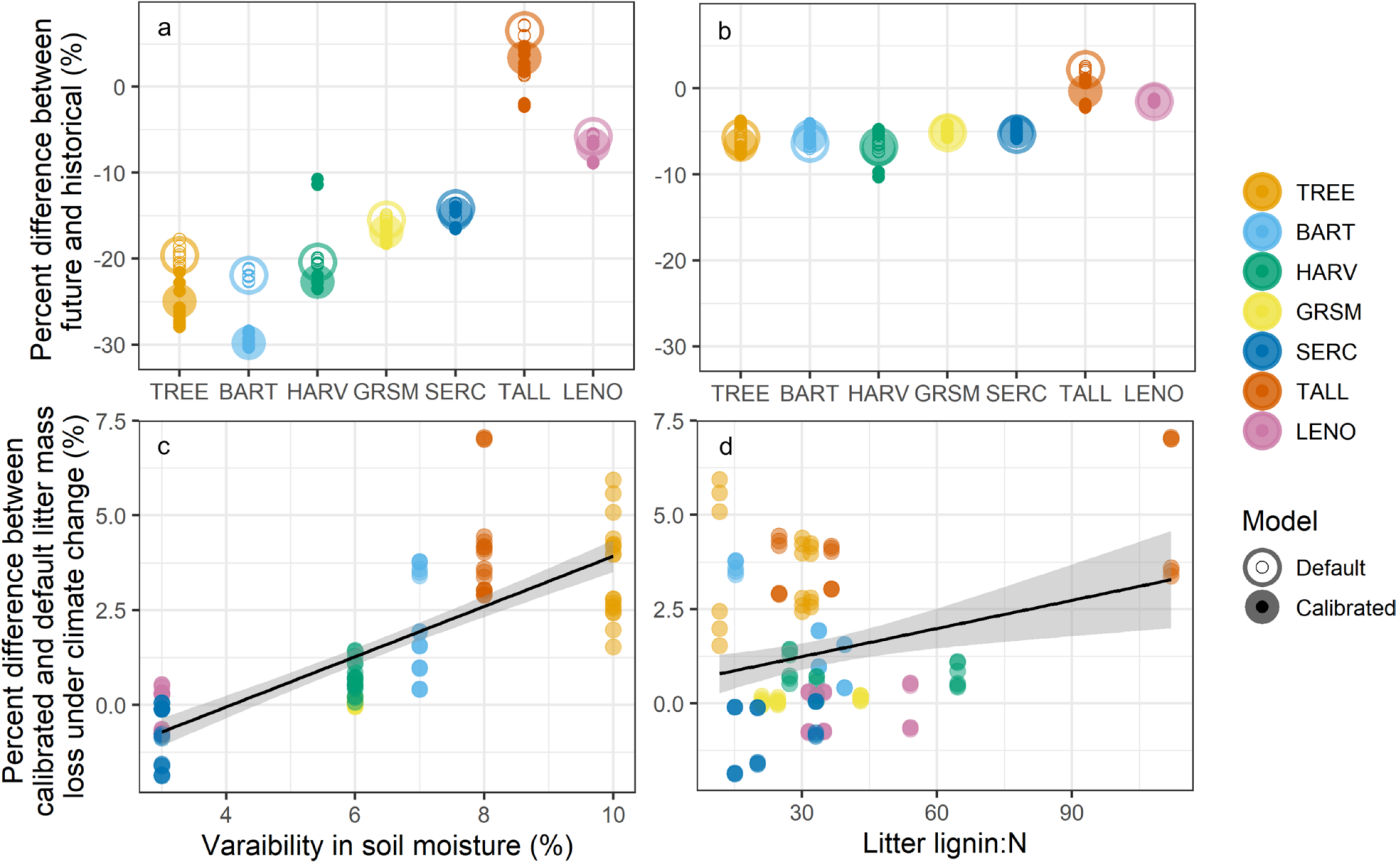
Drivers of the differences between the default and calibrated models under climate change. Plots (a) and (b) show the percent difference in (a) metabolic and (b) structural litter in litterbags between future and historical model runs for the default (open) and calibrated (filled) models across sites (color) for the field litter decomposition time period. Large points represent means for the site for each model, and smaller points represent average percent difference in litter for each litter type and soil moisture combination (*n* = 9) within each site for each model. Plots (c) and (d) show the relationship between the difference in calibrated and default responses to climate change at time points 1 and 2 with (c) variability in soil moisture and (d) litter quality (lignin‐to‐nitrogen ratio). The *y*‐axis for (c) and (d) is the percent difference between the calibrated and default model output for the smaller points in (a) and (b). Sites are ordered from coldest (TREE) to warmest (LENO).

Accelerated rates of litter mass loss under climate change projections were common, regardless of model parameterization, as compared to the historical runs (using 2018–2022 climatology; Figure 5). A notable exception to higher litter mass loss under climate change is for later stage decomposition at TALL, where there is lower litter mass loss under climate change than under historical climate. This is likely because of the very low litter quality at this site for which climate change caused faster loss of the metabolic litter, leaving only slower decomposing structural litter during later stage decomposition, where metabolic and structural litter decomposition have the same temperature sensitivity in MIMICS (Figure 5c; Figure S9). In response to climate change, the calibrated model generally predicted higher litter mass loss than the default model (Figure 5a,b). The calibrated model lost up to 5.4% more litter mass than the default model, based on site‐level averages, under 50 years of climate change, but this effect depended on site. TREE, BART, and TALL, which are also the sites with the lowest annual litterfall, exhibited the highest losses of litter mass under climate change in the calibrated relative to the default model (Figure 5c). This greater mass loss is dominantly through metabolic litter for TREE and BART and through structural litter for TALL (Figure 6a,b; Table S2). We also analyzed the drivers of differences in litter mass loss between the calibrated and default model under climate change (e.g., what variables were associated with higher mass loss under climate change for the calibrated relative to default model). We found lignin:N and soil moisture variability to be consistently significant and positive drivers, regardless of whether they were included with historical, future, or future minus historical litterfall and soil temperature and moisture (Figure 6c,d).

Regardless of the model parameterization, the average effect of climate change on litter mass loss appears to be stronger for the colder sites (Figure 5). This is likely related to stronger increases in soil temperature at colder sites than warmer sites and higher moisture availability in winter at colder sites under the climate change scenario (Table S3; Figure S10). Effect sizes from linear mixed effect models were similar under climate change compared to the historical runs for default and calibrated models, respectively. That is, except for the copiotroph:oligotroph ratio effect size in the calibrated model, which was similar in magnitude but opposite in direction (positive), indicating copiotrophs were more important drivers of decomposition in the calibrated model under climate change (Figure 1; Table 3).

## 4 Discussion

Our work sought to both (1) evaluate how calibrating litter decomposition of a process‐based ecosystem model using empirically important drivers can influence model performance and predictions and (2) serve as a case study for integrating empirical microbial community data into an ecosystem model that represents multiple functional microbial groups. Our results demonstrate that we can calibrate parameters to empirical drivers of litter decomposition while predicting litter decomposition rates equally as well (or better) than with the default parameters. Moreover, we independently validated results from our calibrated model at sites that were not used in model calibration. Despite calibration changing model predictions about the decomposition abilities of copiotrophs and oligotrophs, the default and calibrated models responded similarly to climate change with exceptions at sites with high soil moisture variability and very low litter quality. Similarities between calibrated and default models could be taken to mean that calibration was not necessary. However, calibration provided increased confidence in model outputs because it increased model realism by representing advanced mechanistic understanding, in this case by ensuring the calibrated model was underlain by empirically important drivers of litter decomposition (Knutti and Sedláček 2013). Additionally, at the sites where there were differences in decomposition with the calibrated and default parameters, these differences might indicate that it is quantitatively important to represent empirical drivers in models when assessing potential C cycle–climate feedbacks. Our work also demonstrates an important step toward integrating empirical microbial community data (represented by the copiotroph:oligotroph ratio) into functional model representations of microbial communities, and subsequently, projections of terrestrial ecosystem responses to climate change.

### 4.1 Ecological Dynamics in a Model Calibrated to Empirical Drivers

Ecosystem models can sometimes accurately predict ecosystem dynamics without necessarily representing their underlying drivers (Wieder, Grandy, et al. 2015). While this could be sufficient under steady state conditions, accurately representing ecological processes in models under environmental change may drive alternate responses compared to models without process representation (Wieder et al. 2013; Reich et al. 2014; Sulman et al. 2014, 2017; Guo et al. 2020; Rocci, Cleveland, et al. 2024). For this reason, it is important to ensure process‐based models represent our best empirical understanding. In our work, the observed effect sizes showed that soil moisture was positively related and lignin:N and copiotroph:oligotroph ratios were negatively related to litter decomposition (Figure 1). Given the expected increase in litter mass loss with soil moisture in non‐flooded soils (Prescott 2010), and findings that a positive relationship between soil moisture and litter mass loss might be stronger when considering variation at both the local and regional scale as we did (Bradford et al. 2017), our findings were not surprising. Similarly, higher litter quality (lower lignin or lignin:N) has long been associated with faster decomposition (Meentemeyer 1978; Aerts 1997), and some work suggests litter quality is a stronger driver of litter decomposition than climate (Zhang et al. 2008; Petraglia et al. 2019). The negative relationship between copiotroph:oligotroph and decomposition rates was contrary to our hypotheses, given copiotrophs are defined as having faster growth and, consequently, decomposition (Wieder, Grandy, et al. 2015; Couso et al. 2023). However, given the relatively lignin‐rich nature of temperate leaf litter and the relatively high lignin:N values of litter in this study relative to a global analysis of forest leaf litter (García‐Palacios et al. 2016; global mean = 16.9, this study = 35.2), it is reasonable that oligotrophs caused faster decomposition, given their better ability to decompose structural litter theoretically and in MIMICS. This is further supported by findings of associations between oligotrophic bacteria and litters with higher lignin (Heděnec et al. 2023). Future studies on whether oligotrophs indeed thrive on low quality litter are warranted to determine how generalizable this relationship is across larger spatial extents and over decomposition time frames.

The default model in our study provided relatively similar effect sizes to the observations except that the litter quality effect size was weaker than in the observations and, notably, the effect size of the copiotroph:oligotroph was in the opposite direction as the observations (Figure 1). The latter means MIMICS with the default parameters assumed copiotrophs were the most important decomposers of temperate forest litter, which may be due to the faster decomposition capabilities of copiotrophs in MIMICS. When we calibrated MIMICS to the empirical drivers of decomposition, we improved agreement with field decomposition for both the calibration and independent validation sites (Figures 2 and 3). Parameter changes associated with calibration caused oligotrophs to be more rapid decomposers of metabolic litter and copiotrophs to be slower decomposers of structural litter (Figure 4a,b). This shift allowed for the negative relationship between copiotroph:oligotroph and decomposition in the calibrated model that aligned with observed effect sizes (Figure 1). The parameters that caused these changes to model dynamics are associated with catabolic capacity and microbial turnover, and so our results suggest these microbial traits might be particularly important for litter decomposition, although there is little empirical data on these traits (Supporting Information: Text A). Additionally, the calibrated model simulated a stronger relationship between litter quality and microbial community composition (Figure 4c), amplifying assumptions in MIMICS that structural litter preferentially supports an oligotrophic community and metabolic litter preferentially supports a copiotrophic community. At the extreme end of this, when litter quality was very low, only oligotrophic microbes survived in the calibrated version of MIMICS. This result could lend support to the idea that representing microbial communities in process‐based models is not always necessary since a single microbial pool could adapt to changes in litter quality (Yang et al. 2023). Alternatively, this result could highlight that representing functional groups with specific traits is particularly important in specific ecosystem contexts (e.g., oligotrophs when litter quality is low). Regardless, representing two functional groups of microbes provides ecological realism that improves confidence in our model projections relative to a singular representation of soil microbes (Knutti and Sedláček 2013; Bradford et al. 2016). However, which functional representation of soil microbes in ecosystem models is the most appropriate for a given modeling goal remains unclear. For example, by characterizing the microbial community into copiotrophs and oligotrophs using bacterial data, we are able to represent traits of growth rate and substrate preference for bacteria. Thus, our findings can likely be applied to other temperate forest leaf litter decomposition by bacteria, assuming that growth rate and substrate preference are important for litter decomposition, for which there is evidence in the literature (Goldfarb et al. 2011; Zeng et al. 2019). However, traits and organisms (e.g., fungi) not captured by our microbial grouping are likely important to consider in future work (see Section 4.3).

In our study, the effect sizes of copiotroph:oligotroph ratio for calibrated output of the validation and climate change runs were more positive than the historical runs and observations (Table 3). On one hand, we could assume the effect sizes from the historical calibration (and by extension, the observations) should be representative over space and time (e.g., for the validation sites and climate change runs). With that assumption, the different effect sizes in validation and climate change runs compared to the historical runs might indicate that a structural change (rather than a parametric one) is required to ensure MIMICS always represents oligotrophic microbes as the relatively more important drivers of temperate forest leaf litter decomposition. On the other hand, the different effect sizes could inform testable hypotheses moving forward. For example, we could hypothesize that the influence of copiotrophic microbes on litter decomposition is greater at validation sites due to the higher litter quality at those sites (lignin:N = 5.7–37.1; Table 1). Or, for the climate change runs, we could hypothesize that copiotrophic microbes become more dominant decomposers under warming because their faster decomposition kinetics, relative to oligotrophs, would be amplified due to the temperature sensitivity of decomposition in MIMICS. Hence, our calibration can inform future studies.

### 4.2 Litter Decomposition Response to Future Climate Change

The effect of calibration on responses of litter mass loss to climate change was site‐dependent (Figure 5). At three of the sites we investigated (TALL, BART, and TREE), calibration caused faster litter mass loss under climate change (up to 5% higher), with potential implications for CO_2_ emissions to the atmosphere and soil C formation. According to our statistical analysis, this effect was driven by within‐site soil moisture variability (at TREE and BART) and litter quality (at TALL; Figure 6). The influence of soil moisture variability (e.g., spatial and temporal variation within a site represented with the minimum and maximum soil moisture multipliers) in our results could be due to the fact that, in MIMICS, the fraction of water content in the soil is a multiplier on decomposition rate. Since oligotrophs in the calibrated model were better able to decompose metabolic litter and losses of metabolic, and not structural, litter were driving higher mass loss under climate change for BART and TREE, this effect might be exaggerated at the high end of more variable soil moisture (e.g., the maximum soil moisture multiplier) at these sites. However, we would expect that the lower end of soil moisture (e.g., the minimum soil moisture multiplier) could balance the potential influence of higher soil moisture, so the influence of soil moisture variability remains unclear. Rather, soil moisture variability might be indicating an unmeasured driver or site effects more broadly. While the effect of litter quality can be explained more directly—the calibrated model made oligotrophic microbes better decomposers, in contrast to our hypothesis, and low litter quality increased oligotrophic biomass—it also was clearly driven by low litter quality at the TALL site (Figure 6), again potentially indicating site‐specific effects. For example, the TALL site is unique (compared to our other sites) in its low litter quality, but also it is a very warm site with sandy soils, making it generally unique among the sites represented in our study (Table 1). Thus, site effects beyond soil moisture, litter lignin:N ratio, and copiotroph:oligotroph ratio may be important drivers of litter decomposition that warrant further attention. Further, these site‐specific effects may also highlight interactions between our drivers. While we feel confident that these drivers are not correlated in our dataset (all models had variance inflation factors below 5) and thus our statistical approach was valid, higher water availability has been shown to confer better plant quality (Aerts 1997), and the relative abundance of copiotrophs and oligotrophs has also been shown to be associated with substrate lability (Goldfarb et al. 2011). Thus, a study implementing a similar method to ours could assess these drivers with hierarchical modeling to better capture these interactions. Finally, changes in soil temperature and litterfall are expected to be important drivers of variation in litter decomposition under climate change. However, we do not include within‐site temperature and litterfall variation in our calibration due to lack of data on these metrics, so it is not surprising that they did not influence climate change‐induced litter mass loss in the calibrated vs. default model (Section 3.4). Including within‐site variation in soil temperature and the influence of litterfall amount in future calibrations applied under climate change would help determine whether they are influential in models that integrate these drivers.

Regardless of the parameter set used in the model, litter decomposition at sites with colder mean soil temperatures responded more strongly to climate change in our study (Figure 5). Broader literature on decomposition (including litter and soil organic matter) has long supported stronger climate change responses in colder areas, with higher long‐term Q10s (the increase in activity for a 10°C increase in temperature) of heterotrophic respiration associated with colder temperatures or increased latitude (Lloyd and Taylor 1994; Kirschbaum 1995; Zhou et al. 2009), although this depends on moisture limitation (Aerts 2006). However, literature on litter decomposition specifically is more equivocal. One global study found higher climate change‐induced litter C release in cold places, where temperature increased by the greatest percentage under climate change, as in our study (Chen et al. 2024; Table S3). Another found, in contrast, that litter decomposition responded most positively to warming in warm environments (Liu et al. 2024). The former used SSP scenarios to project climate change, similar to our study, whereas the latter synthesized warming experiments. This suggests other changes in climate change scenarios, such as greater moisture availability in the winter at cold sites (Figure S10), may also drive stronger responses to climate change at cold sites, rather than temperature per se. However, reduced soil moisture is also expected with climate change, and seasonal dryness in temperate forests may negatively interact with warming to reduce litter decomposition (Butenschoen et al. 2011; Reich et al. 2018), so investigating temperature and moisture interactions would be an important future direction. If these expectations of relatively greater decomposition rates at colder sites under climate change do occur, our findings suggest that decomposition rates would get more similar across the eastern US temperate region under climate change as colder sites begin to increase to similar rates as warmer sites and increase overall.

### 4.3 Limitations and Future Work

We believe that our work is a useful starting point for integrating empirical microbial community data into process‐based models. We have demonstrated a method using categorization of bacteria into copiotrophic and oligotrophic groups using phylogeny (Ho et al. 2017; Averill et al. 2021) to capture variation in the trait of microbial growth rate (Walkup et al. 2023). This method has potential limitations, including uncertainty about how finely phylogeny must be defined to be predictive of growth rate (Stone et al. 2023). Alternative methods to determine growth rate, such as quantitative stable isotope probing, would likely provide better estimates for future work (Walkup et al. 2023; Stone et al. 2023). We highlight the challenge of categorizing fungi into copiotrophs and oligotrophs and so identify a need for microbial work to establish whether shifts in copiotrophs and oligotrophs for bacteria reflect similar shifts in fungal communities, which would allow for integration into MIMICS with the same method presented here. Additionally, we highlight needs for evaluation of other soil microbial traits, particularly those relevant to fungi (e.g., Camenzind et al. 2024), for their empirical relevance for decomposition and for methods of integration into models. Specific traits relevant to MIMICS and similar models could include carbon use efficiency, biomass chemistry, biomass and hyphal turnover, and substrate‐specific decomposition rates. Other methods that have incorporated functional microbial information via calibration at the ecosystem scale used enzyme activities and functional gene expression (Li et al. 2019; Gao et al. 2020; Guo et al. 2020; Tao et al. 2024). However, given the wide availability of 16S ribosomal gene sequence data, we believe our method is a meaningful addition to options for incorporating microbial community data into ecosystem models. It is not tractable to represent the vast diversity of soil microbes in soil biogeochemical models, so it is imperative that empiricists and modelers work together to identify the best ways to represent this diversity. Here, we simplify the microbial community into two microbial functional groups, but alternative methods include adding mathematical functions that can represent community processes (Georgiou et al. 2017), using scaling tools from math and physics (Wan and Crowther 2022), or deriving traits from fine‐scale models to use in ecosystem and larger scale models (Karaoz and Brodie 2022; Marschmann et al. 2024). Another useful way forward may be to aim for generalizable microbial parameters that could be used across models (Jian et al. 2024). It is also important to ensure temporal microbial dynamics in process‐based ecosystem models align with those in empirical work, as time‐varying data is rarely used for model validation (Le Noë et al. 2023). In the case of litter decomposition, findings of co‐varying microbial communities with litter quality and environmental factors during decomposition (Matulich et al. 2015; Šnajdr et al. 2011; but see Bray et al. 2012) suggest assessing variability in all of these controls together could be important for ensuring accurate temporal dynamics in process‐based models.

Beyond providing an example of how to integrate empirical microbial community data into an ecosystem scale model, we also present a novel method of calibration. By combining traditional empirical statistical methods with Monte Carlo parameter estimation, we demonstrate a way to bridge empiricism and process‐based modelling when observations cannot be directly plugged into models as parameters, which is commonly the case for soil microbial parameters (Wan and Crowther 2022). Additionally, our work highlights that parameterizing models to predict not just for the right answers but for the right reasons is important for ensuring confidence in modelled results (Bradford et al. 2016) and, in some cases, could modify future C cycle–climate feedbacks. Ensuring confidence in our model projections is key for ensuring the science in our models is certain enough to support decision‐making around climate action and adaptation.

While our work captures several dominant drivers of litter decomposition, other drivers may be playing a role in our results and are worth investigating for their representation in process‐based models. Temperature is a key driver of litter decomposition, with its effects potentially mediated by soil moisture (Petraglia et al. 2019). In our work, we lacked within‐site measurement of soil temperature to represent this aspect of climate in our calibration, and site‐level temperature had a similar effect on decomposition as within‐site soil moisture (Figure S2). In other datasets where temperature data are available at the fine scale or uniquely influence decomposition, temperature should be assessed as an additional climate driver to soil moisture. Soil nutrient availability and soil fauna are also commonly cited drivers of litter decomposition (Hobbie 2015; García‐Palacios et al. 2013). Future work could employ a similar method as in our work to a soil biogeochemical model with coupled C and N, calibrating each site to soil N availability, or could use the model as a tool for disentangling litter quality and soil nutrient availability feedbacks by changing drivers independently and together. Since our litterbags allowed for access by fauna, different faunal communities across and within sites may have directly or indirectly, by changing the microbial communities, altered litter decomposition (Frouz 2018) but we lack the data to test this and model representation of fauna. Indeed, few process‐based models include representation of fauna (Grandy et al.^2016^), but as fauna are important drivers of litter decomposition, assessing their roles in process‐based models would be useful future work.

## 5 Summary

Given the empirical evidence suggesting that the microbial community is an important driver of litter decomposition, and the ability of ecosystem and Earth system models to now represent microbial communities, we sought to evaluate (1) the influence of representing empirical drivers of litter decomposition, including microbial community (represented as the copiotroph:oligotroph ratio), for ecosystem model performance and predictions and (2) the potential for integrating microbial composition data into an ecosystem model with functional representation of microbial communities. We show that we are able to reasonably represent three key empirical drivers of litter decomposition as proxies for climate, litter quality, and microbial community without compromising model performance. Further, representing empirical drivers predicted higher litter mass loss under climate change at three of our sites, with implications for C cycle–climate feedbacks. We also provide a useful case study for assimilating empirical bacterial community data into functional microbial groups in an ecosystem model. Our study demonstrates that adding ecological realism to process‐based models does not necessarily incur a loss in model performance and that including microbial community data in these models in functional ways may provide more confidence in our models' abilities to predict ecosystem processes under global environmental change.

## Author Contributions

**Katherine S. Rocci:** conceptualization, data curation, formal analysis, visualization, writing – original draft. **Derek Pierson:** formal analysis, methodology, software, writing – review and editing. **Fiona V. Jevon:** data curation, methodology, project administration, writing – review and editing. **Alexander Polussa:** data curation, formal analysis, writing – review and editing. **Angela M. Oliverio:** data curation, formal analysis, writing – review and editing. **Mark A. Bradford:** conceptualization, funding acquisition, project administration, writing – review and editing. **Peter B. Reich:** conceptualization, funding acquisition, writing – review and editing. **William R. Wieder:** conceptualization, funding acquisition, methodology, software, supervision, writing – review and editing.

## Conflicts of Interest

The authors declare no conflicts of interest.

## Supporting information


Data S1.

## Data Availability

The data and code that support the findings of this study are openly available in Zenodo at https://doi.org/10.5281/zenodo, Dryad at https://doi.org/10.5061/dryad.5hqbzkhg6, and NCBI SRA under Bioproject PRJNA1198092. CESM 2.1.2 code was obtained from the University Corporation for Atmospheric Research (UCAR) at https://www.cesm.ucar.edu/models/cesm2.
