# Supplementary material for: Integrating Microbial Community Data Into an Ecosystem‐Scale Model to Predict Litter Decomposition in the Face of Climate Change
Source: Glob Chang Biol. 2025 Jul 17;31(7):e70352. doi: 10.1111/gcb.70352 (PMC12268835; doi:10.1111/gcb.70352)
Supplement: Supplementary file 1 — Data S1. [file GCB-31-e70352-s001.pdf]

Supplemental Material for:

Integrating microbial community data into an ecosystem-scale model to predict litter decomposition in the face of climate change

Katherine S. Rocci[1, 2], Derek Pierson[3], Fiona V. Jevon[4], Alexander Polussa[4], Angela M. Oliverio[5], Mark A. Bradford[4], Peter B. Reich[2, 6], William Wieder[1, 7]

[1]Institute of Arctic and Alpine Research, University of Colorado, Boulder, CO, 80309

[2]Institute for Global Change Biology, University of Michigan, Ann Arbor, MI, 48109

[3]Rocky Mountain Research Station, United States Forest Service, Boise, ID 83702

[4]The Forest School, Yale School of the Environment, Yale University, New Haven, CT, 06511, USA

[5]Biology Department, Syracuse University, Syracuse, NY 13244

[6]Department of Forest Resources, University of Minnesota, St. Paul, MN 55108

[7]Climate and Global Dynamics Laboratory, National Center for Atmospheric Research, Boulder, CO 80307

Correspondence to: Katherine S. Rocci (katie.rocci@colorado.edu)

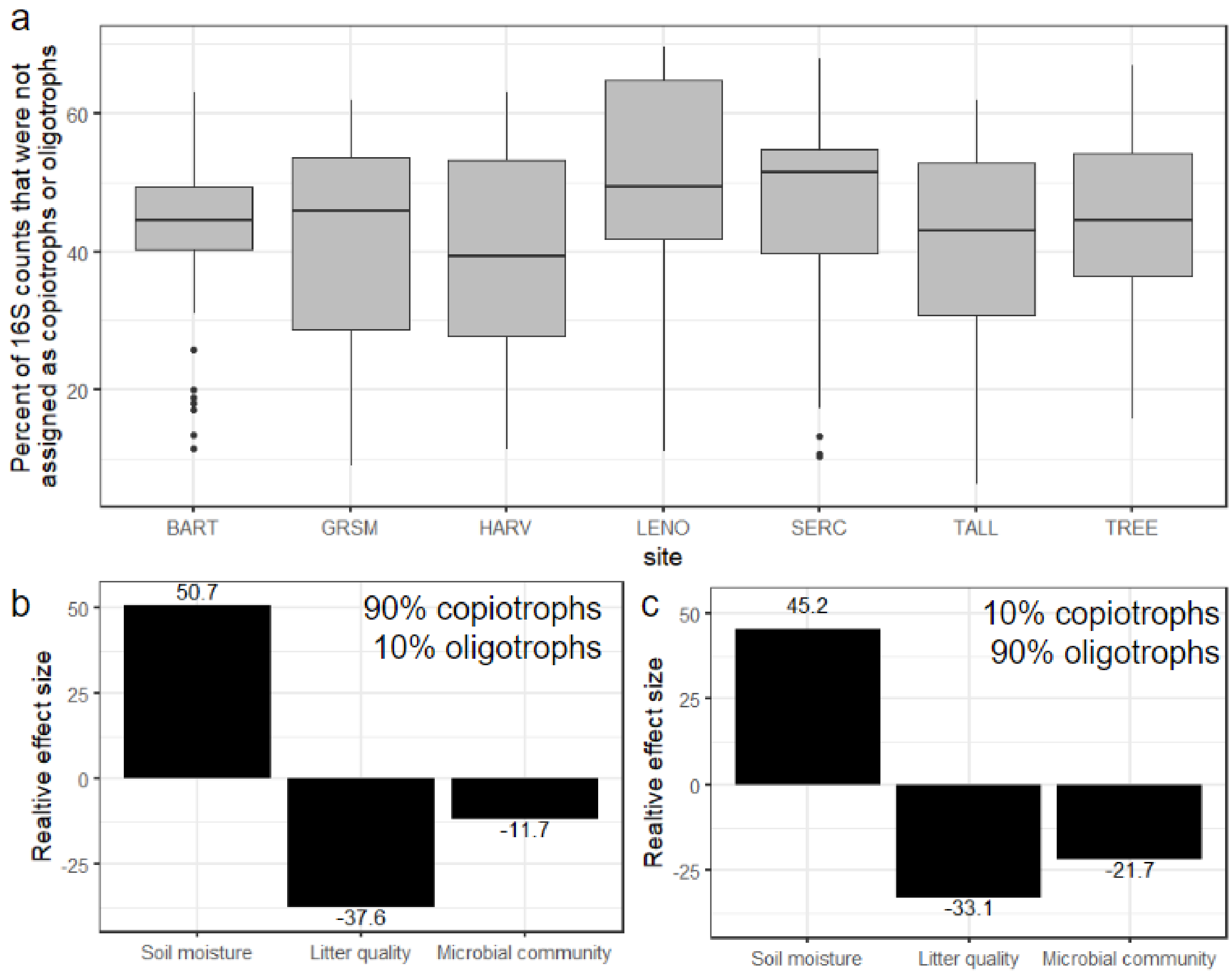

Supplementary Figure 1: The (a) percent of counts within a sample that were not assigned to copiotrophs or oligotrophs for each site and the (b) minimum, where 90% of unassigned taxa are assigned to copiotrophs and 10% to oligotrophs, and (c) maximum, where 10% of unassigned taxa are assigned to copiotrophs and 90% to oligotrophs, effect sizes for the copiotroph:oligotroph ratio for the observed linear mixed effect model predicting litter mass loss.

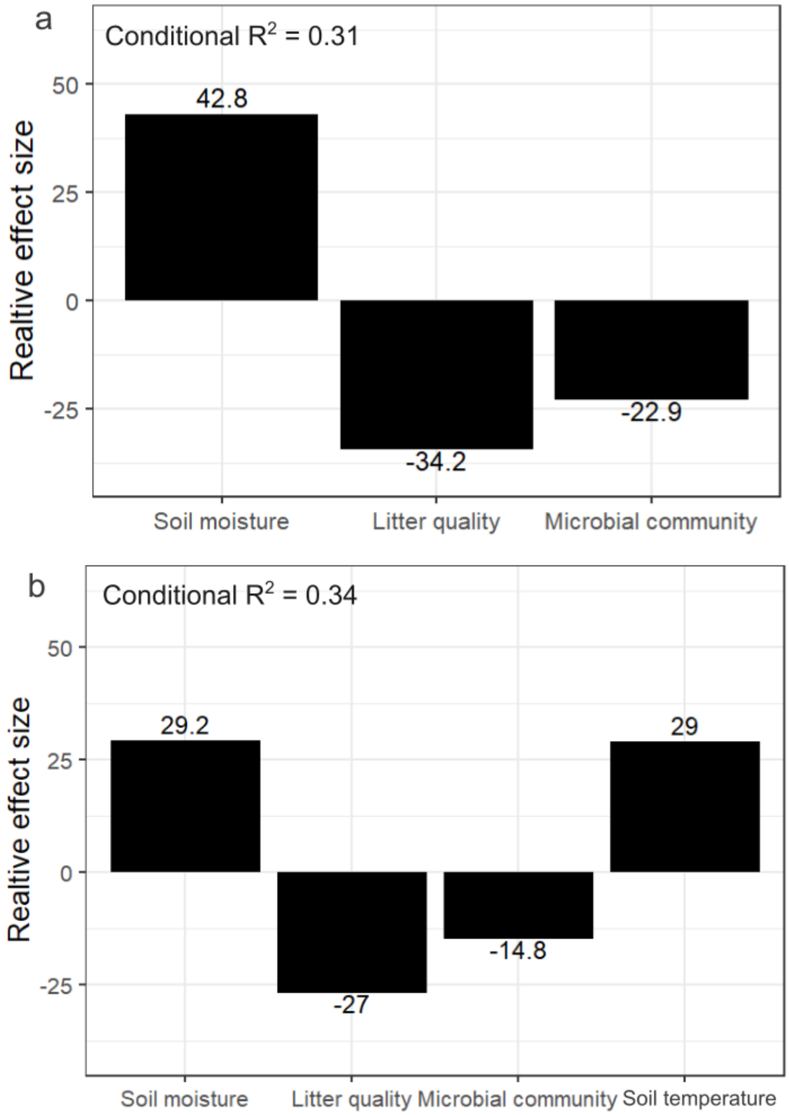

Supplementary Figure 2: Comparison of empirical relative effect sizes (a) without and (b) with site-level mean temperature (from Table 1) included in the statistical model predicting litter decomposition and associated conditional R^2 values for each model.

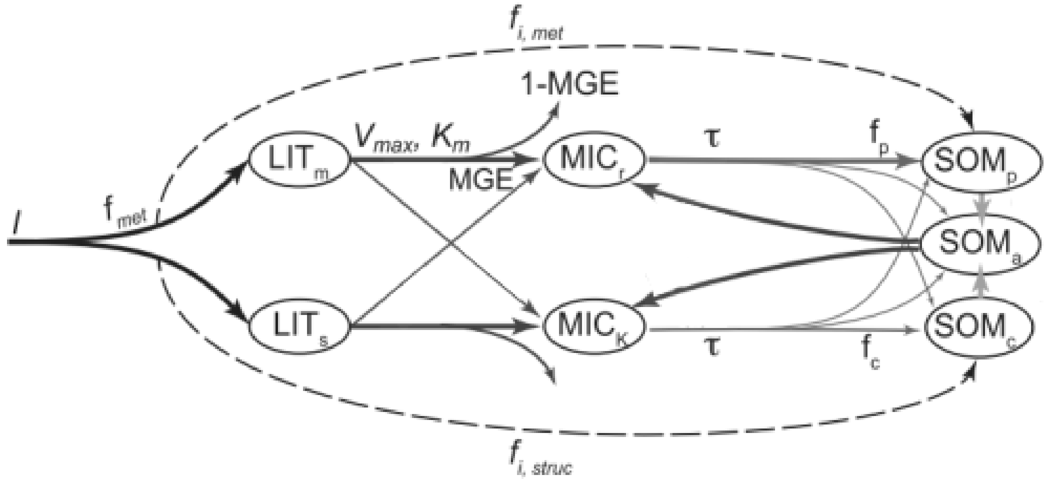

Supplementary Figure 3: The MIcrobial-MIneral Carbon Stabilization (MIMICS) model wiring diagram. MIMICS separates litter into a metabolic and structural litter pool (LITm and LITs) using a fraction of metabolic litter (f_{met}) calculation dependent on the lignin:N of incoming litter. LITm is preferentially decomposed by a copiotrophic microbial group (MICr) and LITs by an oligotrophic microbial group (MICk), which subsequently contribute C preferentially to physically-protected and chemically-protected soil organic matter pools (SOMp and SOMc), as well as to the available SOM pool (SOMa), from which microbes can also assimilate C. Fluxes between pools are controlled by parameters like Vmax and Km, which determine the max speed of decomposition, and microbial growth efficiency (MGE) and tau (τ), which determine the proportion of assimilated C and the rate of microbial turnover, respectively.

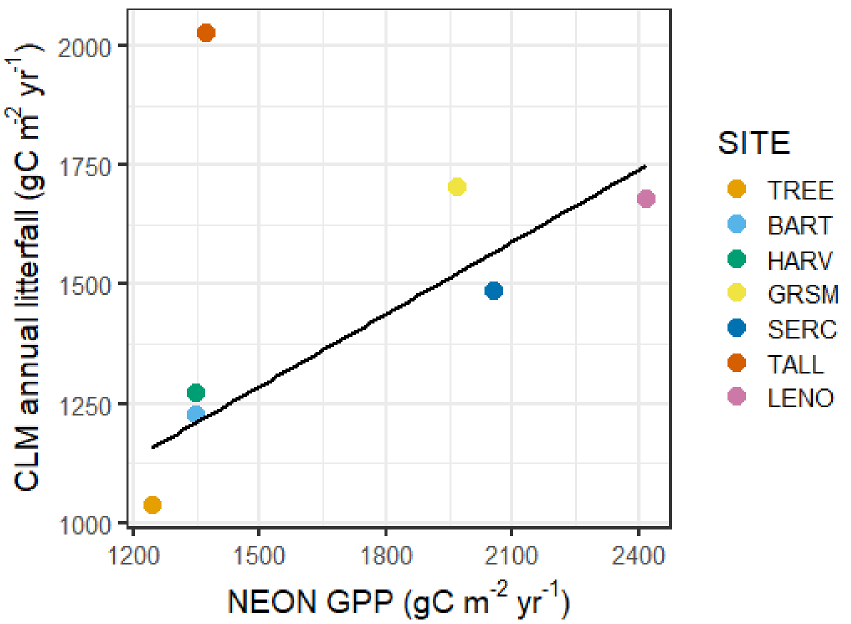

Supplementary Figure 4: Comparison of modeled annual litterfall data from the Community Land Model (CLM) and gross primary production (GPP) estimates from National Ecological Observatory

Network (NEON) flux tower measurements averaged over 2018-2021. The linear relationship (black line) between these for all sites except TALL was used to correct TALL annual litterfall data.

Supplementary Table 1: MIMICS parameters used in this study before modifications to beta and Vmax, and before calibration.

Parameter	Value	Description
Vslope	exp(TSOI*0.063*Vint)	regression coefficient for Vmax
Vint	exp(TSOI*Vslope*5.47)	regression coefficient for Vmax
aV	0.000008	tuning coefficient on Vmax
Kslope	exp(TSOI*(0.025, 0.035, 0.025)*Kint)[a]	regression coefficient for Km
Kint	exp(TSOI*Kslope*3.19)	regression coefficient for Km
aK	10	tuning coefficient on Km
vMOD	10, 2, 10, 3, 3, 2[b]	modifier on Vmax fluxes
kMOD	8, 2, 4, 2, 4, 6[b]	modifier on Km fluxes
KO	6	modifier on Km for oxidation of SOMc
CUE	0.55, 0.25, 0.75, 0.35[c]	microbial carbon use efficiency
tau_r	0.00052*exp(0.3*fMET)	copiotroph microbial biomass turnover rate
tau_K	0.00024*exp(0.1*fMET)	oligotroph microbial biomass turnover rate
fPHYS_r	0.3*exp(1.3*fCLAY)	proportion of copiotrophic turnover to SOMp
fPHYS_K	0.2*exp(0.8*fCLAY)	proportion of oligotrophic turnover to SOMp
fCHEM_r	0.1*exp(-3*fMET)*1	proportion of copiotrophic turnover to SOMc
fCHEM_K	0.3*exp(-3*fMET)*1	proportion of oligotrophic turnover to SOMc

fSOM_p	1.5e-5*exp(-1.5*fCLAY)	desorption rate of SOMp to SOMa
PHYS_scalar	2*exp(-2*sqrt(fCLAY))	Scalar for texture effects on SOMp
FI	0.05, 0.05[d]	fraction of litter inputs transferred to SOM
fmet_p	1*(0.85-0.013*LIG_N)	partitioning of litter inputs to LITm

[a]decomposition for LITm, LITs, and SOMa ; [b]LITm, LITs, and SOMa to MICr and LITm, LITs, and SOMa to MICK ; [c]LITm/SOMa and LITs to MICr and LITm/SOMa and LITs to MICK ; [d]for metabolic and structural litter inputs entering SOMp and SOMc respectively

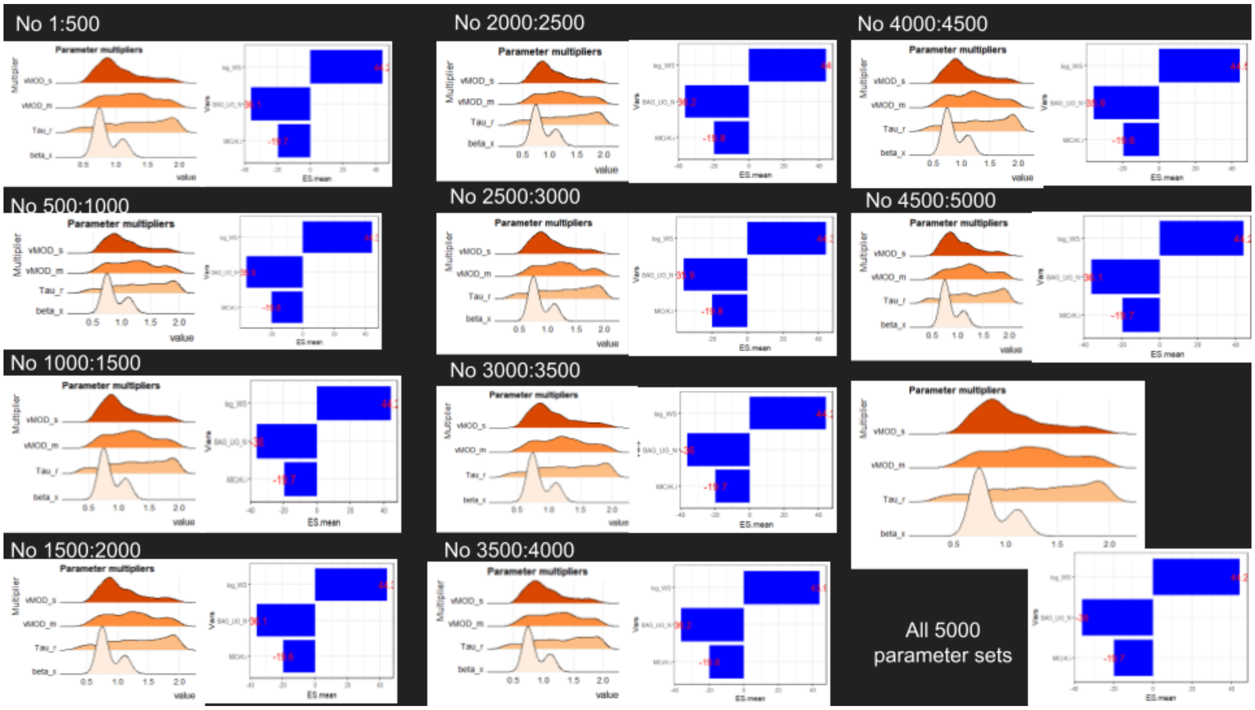

Supplemental Figure 5: Sensitivity analysis to ensure there were a sufficient number of random parameter sets. We removed random parameter sets in groups of 500 (e.g., 10% of the parameter sets) and visually compared the distribution of parameter multipliers that passed initial filtering (the first two steps described in the methods) using the left plots and the average effect sizes of those parameter sets using the right plots to those with all 5000 parameter sets (bottom left plots). We also analyzed how removing these same groups of 500 affected the R^2 and RMSE of the modeled litter mass loss of the top three parameter sets as compared to observations. However, since all three top parameter sets were in the 4500-5000 parameter group, the only modification was for this group. For the 4500-5000 group we calculated the R^2 and RMSE of the next two best parameter sets and these only decreased by 0.01 and increased by 0.1, respectively.

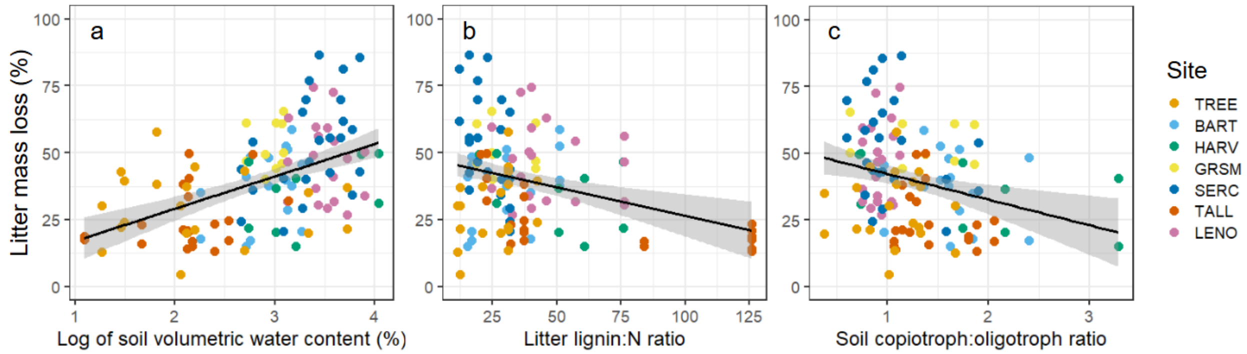

Supplementary Figure 6: Relationship between litter mass loss and (a) log of soil volumetric water content, (b) litter lignin:nitrogen (N) ratio, and (c) soil copiotroph: oligotroph ratio for the empirical data, colored by site. Linear relationships are depicted as black lines on plots.

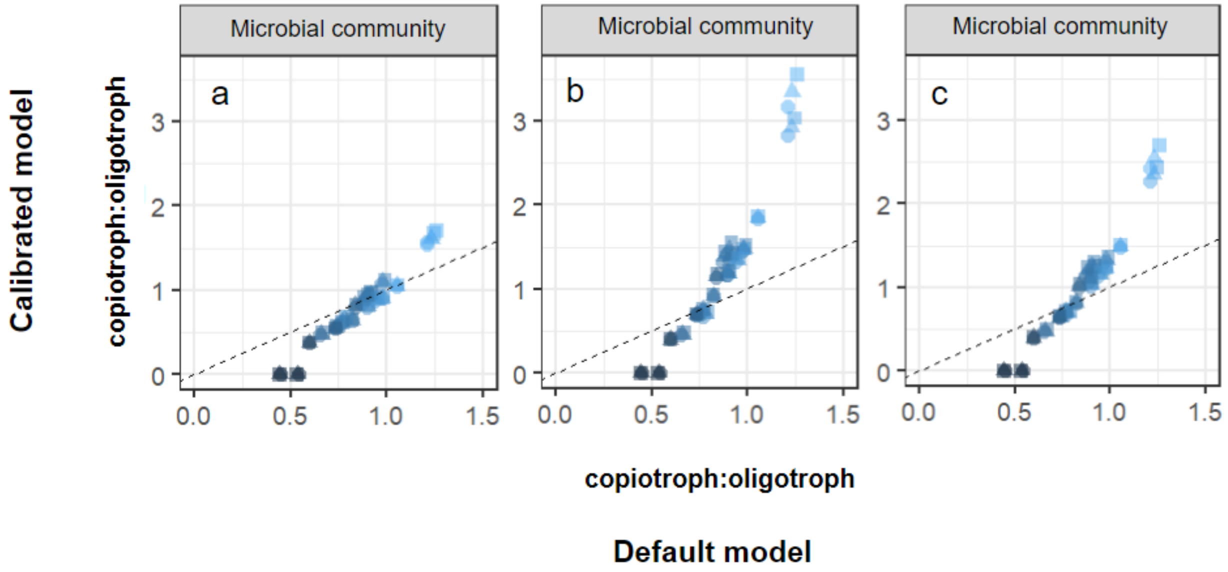

Supplementary Figure 7: Comparison of the microbial community (the copioptroph:oligotroph ratio) in the default and calibrated models for the three top parameter sets (a, b, and c). Dashed line depicts the 1:1 line.

Supplementary Text A: Discussion of specific parameter changes associated with calibration.

The three different parameter sets used in the calibrated model are broadly similar but parameter set 1 has a Tau_r multiplier that is greater than 1, whereas the other two parameter sets have a Tau_r multiplier less than 1, indicating higher and lower copiotrophic turnover, respectively (Table 2). The beta and Tau_r multipliers are linearly related across the three parameter sets. High beta multipliers (greater than 0.742) appear to be associated with high Tau_r multipliers (greater than 1; Supplementary Fig. 7), but the beta mulitpliers always remain under 1 suggesting a smaller effect of density dependence on turnover is best for representing our observational data. The relationship between beta and Tau_r suggests a tradeoff is occurring within the MIMICS structure.

In all three of the best parameter sets, model calibration increased rates of metabolic litter decomposition and decreased rates of structural litter decomposition (Table 2). Specifically, oligotrophs

increased and copiotrophs decreased their decomposition rates of metabolic and structural litter, respectively. Notably, changes to max decomposition rate of metabolic and structural litter (vMODm and vMODs in Table 2) were not applied differently for copiotrophs and oligotrophs, suggesting this microbial group specific outcome is the result of interactions between all four parameters that were changed in the calibrated model. In contrast to consistent changes in the vMOD parameters, the relationship between litter quality and copiotrophic turnover (tau_r parameter) both increases and decreases depending on the parameter set used (Table 2). There appears to be a tradeoff between the tau_r and beta (e.g., the density-dependent effect on turnover) parameters where small increases in beta lead to large increases in tau_r. Practically, this means that as microbial turnover gets more sensitive to density of the microbial population, the effect of litter quality is also greater on copiotrophic turnover. This would cause higher turnover particularly when litter quality is high and consequently copiotrophic biomass is increased, overall potentially stabilizing copiotrophic biomass. More broadly, this tradeoff highlights the large uncertainty in microbial parameters in microbially-explicit models, especially those related to microbial anabolism, which often need to be determined by fitting data and not through direct measurement (Wan and Crowther, 2022). Since adding a density dependent control on microbial turnover (beta parameter used here) is intended to create more realistic microbial dynamics (Georgiou *et al.*, 2017), determining appropriate values for, and interactions between, this parameter and other parameters associated with microbial growth rates and turnover is an important direction for future research.

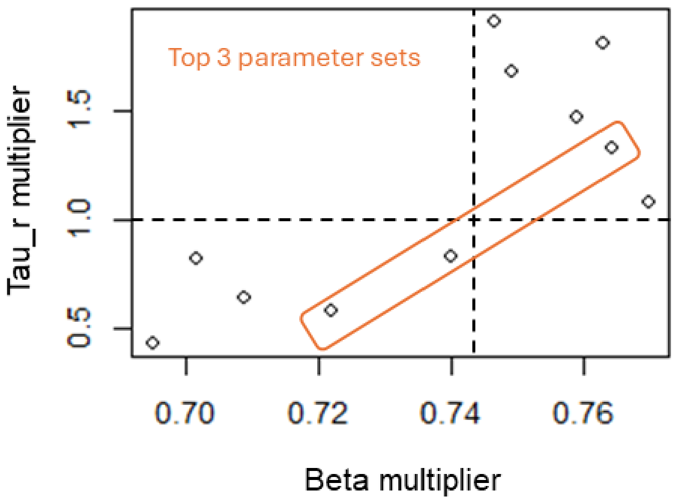

Supplementary Figure 8: Relationship between the beta and tau_r multipliers in the top parameter sets with RMSE<5.8% where at least one microbial pool is maintained. Dotted lines depict apparent tradeoff in these two multipliers and the orange box is highlighting the top 3 parameter sets that were used in the calibrated model.

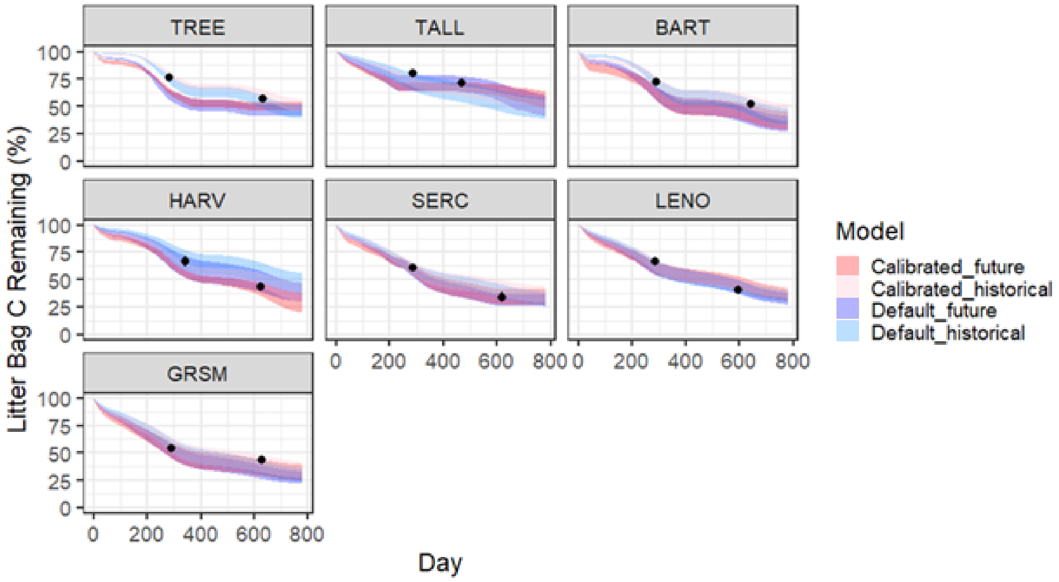

Supplementary Figure 9: Decomposition dynamics at each site (facets) for the default (blues) and calibrated (reds) parameter sets for the historical (2018-2022 climatology; lighter tones) and future (2072-2074; darer tones) time periods. Black points represent observations from 2021-2023 field campaign for time points 1 and 2.

Supplementary Table 2: Mean percent difference (%) in future and historical values for pools and fluxes in the MIMICS model under the default (D in the M column) and calibrated (C in the M column) models. LITBAGm, LITBAGs, LITm, LITs, MICr, MICk, SOMp, SOMc, and SOMa denote the mean percent difference in future vs historical runs across the field decomposition time in the C pools of metabolic litter in litterbags, structural litter in litterbags, metabolic litter, structural litter, copiotrophic microbes, oligotrophic microbes, physically-protected soil organic matter, chemically-protected soil organic matter, and available soil organic matter, respectively. SDR_rm, SDR_rs, SDR_kn, and SDR_ks denote the mean percent difference in future vs historical runs across the field decomposition time for the specific decomposition rate of metabolic litter by copiotrophs, structural litter by copiotrophs, metabolic litter by oligotrophs, and structural litter by oligotrophs, respectively.

SITE	M	LIT BAGm	LIT BAGs	LITm	LITs	MICr	MICk	SOMp	SOMc	SOMa	SDR_ rm	SDR_ rs	SDR _km	SDR _ks
TREE	D	-19.6	-5.7	-5.0	0.1	2.2	14.2	25.4	5.7	-2.3	-4.4	-9.2	9.4	6.3
	C	-24.9	-6.5	-6.5	-5.9	10.0	28.5	26.3	0.6	-10.1	-2.6	-5.1	19.9	24.5
BART	D	-21.9	-6.3	-11.6	-13.8	6.8	8.0	15.4	-14.2	-15.1	6.3	22.2	8.7	24.2
	C	-29.8	-5.6	-11.5	-16.8	4.7	9.1	14.1	-17.2	-17.5	9.6	34.7	0.9	27.6
HARV	D	-20.4	-6.7	-10.6	-13.4	8.7	8.3	17.3	-15.2	-15.7	6.8	26.9	5.4	23.2
	C	-22.7	-7.1	-7.0	-16.1	7.1	8.5	16.1	-18.8	-18.1	10.2	46.5	2.2	18.5

GRSM	D	-15.5	-5.2	-7.5	-6.7	6.3	7.4	12.1	-4.9	-7.9	0.5	6.1	4.1	7.8
	C	-16.9	-5.0	-8.7	-9.6	7.3	11.8	12.2	-7.4	-10.4	-0.5	4.5	9.6	11.1
SERC	D	-14.2	-5.3	-10.5	-6.3	4.6	11.1	17.6	-2.4	-8.7	0.3	2.0	9.0	9.2
	C	-14.8	-5.0	-13.9	-9.8	9.0	20.4	18.3	-5.8	-13.6	-1.2	-0.4	20.4	19.0
TALL	D	6.6	2.2	3.8	8.3	4.5	2.9	14.7	9.3	8.0	-1.9	-3.1	-0.3	-7.9
	C	3.3	-0.3	-0.8	3.6	5.6	2.5	15.8	5.6	2.7	-14.3	-22.6	-7.7	-12.4
LENO	D	-5.8	-1.5	-0.5	-0.4	7.0	5.7	10.4	-0.8	-2.3	0.7	6.3	3.2	5.2
	C	-6.8	-1.4	-0.6	-2.7	11.3	6.1	10.3	-3.1	-4.8	0.4	10.3	4.3	4.9

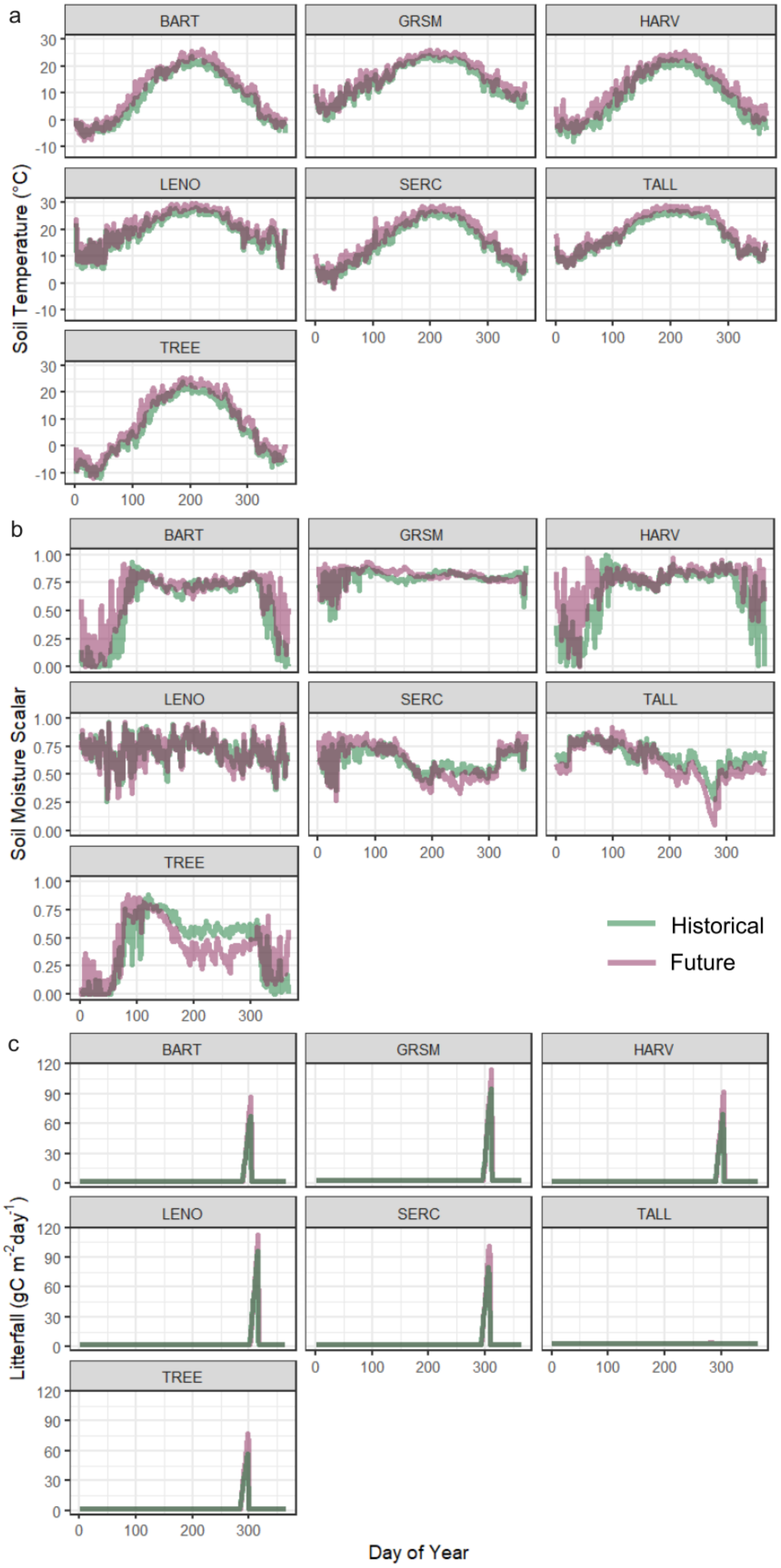

Supplementary Figure 10: Differences in (a) soil temperature, (b) moisture (as depicted by the soil moisture scalar), and (c) litterfall under future compared to historical model runs.

Supplementary Table 3: Differences in future and historical site data used to force MIMICS model in this study. Litterfall, soil temperature, and soil moisture (e.g., the water scalar) were retrieved from Community Land Model output.

SITE	Historical annual litterfall (g C/m2/yr)	Future annual litterfall (g C/m2/yr), *percent change*	Historical soil temperature (°C)	Future soil temperature (°C), *percent change*	Historical water scalar	Future water scalar, *percent change*
BART	613.0	716.5<br>*+17%*	8.5	10.7<br>*+26%*	0.55	0.63<br>*+15%*
GRSM	853.4	954.4<br>*+12%*	14.7	16.7<br>*+14%*	0.80	0.82<br>*+3%*
HARV	637.1	758.6<br>*+19%*	8.9	12.0<br>*+35%*	0.66	0.76<br>*+15%*
LENO	841.1	929.6<br>*+11%*	19.1	21.0<br>*+10%*	0.71	0.70<br>*-1%*
SERC	744.2	865.2<br>*+16%*	14.8	16.9<br>*+14%*	0.62	0.62<br>*0%*
TALL	608.3	694.6<br>*+14%*	18.1	19.7<br>*+9%*	0.66	0.60<br>*-9%*
TREE	518.8	636.6<br>*+23%*	6.8	9.3<br>*+37%*	0.44	0.42<br>*-5%*

References

Georgiou, K., Abramoff, R. Z., Harte, J., Riley, W. J. & Torn, M. S. Microbial community-level regulation explains soil carbon responses to long-term litter manipulations. Nature Communications 8, 1223 (2017).

Wan, J. & Crowther, T. W. Uniting the scales of microbial biogeochemistry with trait-based modelling. Functional Ecology 36, 1457-1472 (2022).
